# Peptide Isolation *via* Spray Drying: Particle Formation, Process Design and Implementation for the Production of Spray Dried Glucagon

**DOI:** 10.1007/s11095-020-02942-5

**Published:** 2020-12-14

**Authors:** Frederik J. S. Doerr, Lee J. Burns, Becky Lee, Jeremy Hinds, Rebecca L. Davis-Harrison, Scott A. Frank, Alastair J. Florence

**Affiliations:** 1 EPSRC CMAC Future Manufacturing Research Hub, Technology and Innovation Centre, 99 George Street, Glasgow, G1 1RD UK; 2 Strathclyde Institute of Pharmacy & Biomedical Sciences (SIPBS), University of Strathclyde, Glasgow, G4 0RE UK; 3 Small Molecule Design and Development, Eli Lilly and Company, Indianapolis, IN 46221 USA; 4 Eurofins Lancaster Laboratories PSS, Indianapolis, IN 46221 USA

**Keywords:** droplet drying, peptide formulation, process development, psychrometric process model, spray drying

## Abstract

**Purpose:**

Spray drying plays an important role in the pharmaceutical industry for product development of sensitive bio-pharmaceutical formulations. Process design, implementation and optimisation require in-depth knowledge of process-product interactions. Here, an integrated approach for the rapid, early-stage spray drying process development of trehalose and glucagon on lab-scale is presented.

**Methods:**

Single droplet drying experiments were used to investigate the particle formation process. Process implementation was supported using in-line process analytical technology within a data acquisition framework recording temperature, humidity, pressure and feed rate. During process implementation, off-line product characterisation provided additional information on key product properties related to residual moisture, solid state structure, particle size/morphology and peptide fibrillation/degradation.

**Results:**

A psychrometric process model allowed the identification of feasible operating conditions for spray drying trehalose, achieving high yields of up to 84.67%, and significantly reduced levels of residual moisture and particle agglomeration compared to product obtained during non-optimal drying. The process was further translated to produce powders of glucagon and glucagon-trehalose formulations with yields of >83.24%. Extensive peptide aggregation or degradation was not observed.

**Conclusions:**

The presented data-driven process development concept can be applied to address future isolation problems on lab-scale and facilitate a systematic implementation of spray drying for the manufacturing of sensitive bio-pharmaceutical formulations.

**Supplementary Information:**

The online version contains supplementary material available at 10.1007/s11095-020-02942-5.

## Introduction

Techniques for the isolation of peptide-based systems are of considerable interest for the development of novel pharmaceutical peptide products [1, 2]. Freeze drying and spray drying are often methods of choice for the solidification of unstable or sensitive bio-pharmaceutical formulations. Freeze drying tends to show less favourable productivity and costs in comparison to spray drying, which is a rapid drying process with capabilities for high product throughput [3]. Operating conditions must be carefully selected during process development to avoid thermal damage due to exposure of the material to excessive drying temperatures or mechanical damage caused by shear stress during the pumping of liquid feed and atomisation in the spray nozzle. Despite these process risks, the rapid drying kinetics and the evaporative cooling effect allow the processing of heat sensitive materials. Spray drying has been successfully utilised for the production of bio-pharmaceutical formulations containing peptides, proteins and related heat-sensitive bio-pharmaceutical products [4–7]. For particle engineering applications, spray drying enables the direct control of product properties including residual moisture as well as particle size and shape, important for stability and performance [8]. Spray drying of peptide-based systems typically includes the use of stabilizers to protect against peptide denaturation during production and to improve storage stability. Carbohydrates are often employed as excipients as they can preserve the protein’s active conformation via preferential exclusion, water replacement and glass immobilization mechanisms [9–11].

At lab-scale, available drying times are often limited due to the short residence times, which require high atomization air flow rates and/or high drying temperatures to achieve stable dry particles. A design-of-experiment (DoE) approach is often used to assess and identify suitable operating conditions [6, 12, 13]. However, a full-factorial design of relevant accessible process parameters is material and time intensive. Process implementation can be assisted using modelling approaches in order to identify promising conditions or avoid adverse process regimes. These models can be based on empirical correlations between selected, independent process variables and measured product properties or derived from first-principles [14–16]. In both cases, experimental data are imperative to quantify process-product dependencies and for model validation.

Process Analytical Technology (PAT) is used to analyse, monitor and control pharmaceutical manufacturing processes. Its use is highlighted by regulatory agencies encouraging manufacturers to integrate PAT during process implementation [17]. For spray drying, the use of PAT has been demonstrated to measure conditions of the drying gas such as its temperature and humidity levels through thermo-hygrometers as well as to capture information on particle properties such as their size distribution with in-line laser diffraction [18, 19] or the material’s solid state attributes employing Raman spectroscopy [20]. Despite its utility, PAT is often not considered for lab-scale applications, relying solely on an off-line characterisation of the product.

Single droplet drying (SDD) experiments have been used to investigate the drying and solidification of solutions, suspensions or melts on a single droplet scale. A popular container-less platform to perform SDD experiments is acoustic levitation [21–23]. Despite the comparatively large droplet sizes and slower drying kinetics, information on droplet evaporation and particle formation can inform spray drying models and support process development [24–26]. Applications of acoustic levitation in combination with micro-X-ray tomography (micro-XRT) aim for a better understanding of the particle formation process, linking formulation parameters and the observed drying kinetics to the final particle morphology and internal micro-structure [21]. In this context, the use of micro-XRT allows the non-destructive extraction of a wide range of quantitative descriptors related to the particle size, shape and porosity [21, 27].

Here we report the successful implementation of a spray drying process for the isolation of a peptide-based model system containing glucagon (GLUC). GLUC is a single-chain polypeptide with 29 amino acids [28] and a commercial pharmaceutical hormone used against insulin-induced hypoglycemia [29]. GLUC was selected as a model peptide with well-documented aggregation pathways [30, 31]. The aggregation mechanism and its kinetics are influenced by various factors including pH, concentration, temperature and hydrodynamics [30–32]. The hydration of peptides or larger proteins affect their folding, stability, dynamics and function [33]. Organic solvents such as ethanol can act as chaotropic agents, disrupting the hydrogen bonding network of the peptide hydration shell and weakening hydrophobic intra-molecular interactions [34–36]. This can lead to a chaotropic solvational behaviour which inhibits ordered fibril formation of proteins, as observed for insulin at ethanol-concentrations of more than 10 wt% [37, 38]. Trehalose (TRE) is a non-reducing sugar and is a common stabilizing excipient in peptide-based systems [39]. TRE is generally preferred over sucrose as it has a higher glass transition temperature (*T*_*g*_) of 115°C compared to *T*_*g*_ of 74°C for sucrose [40]. TRE was used to produce formulated GLUC-powders.

**Fig. 1:**
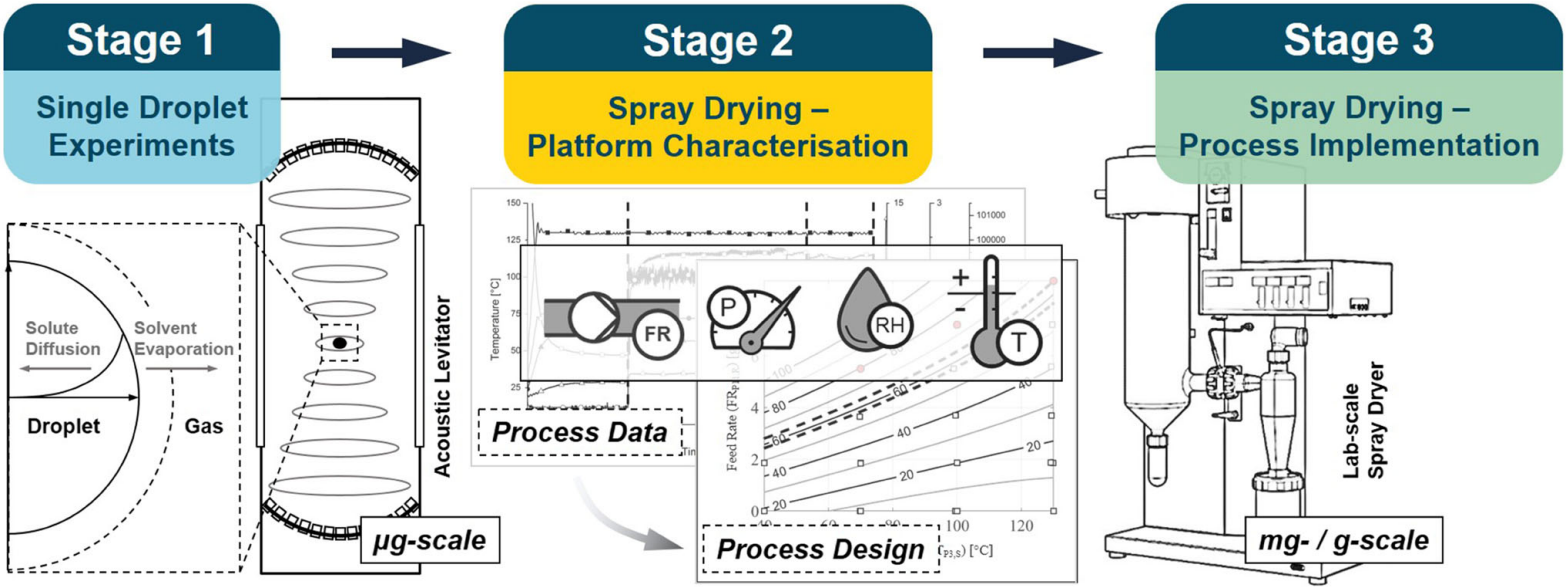
Schematic of the spray drying process development strategy consisting of single droplet drying (SDD) experiments, a characterisation of the spray drying platform and the final process implementation step.

The experimental strategy consists of single droplet drying (SDD) experiments, a characterisation of the spray drying platform and the final process implementation step (Fig. 1). Whilst these have been described individually in previous publications, this study demonstrates their applicability as part of a combined spray drying process development approach on lab-scale. SDD experiments and micro-XRT were used to investigate the particle formation mechanism and quantify properties related to the final particle 3D size and shape. An inexpensive gas sensor was adapted and employed for in-line analysis of the exhaust gas, measuring local temperature, relative humidity and absolute pressure. The collected information was used to evaluate accessible drying conditions during spray drying, which were identified within a psychrometric process model based on heat- and mass-balance considerations for the system. Primary objective for successful process implementation was a high product recovery of isolated active peptide material. Off-line characterisation of the spray dried powders linked product to process conditions assessing particle engineering aspects related to size, morphology, solid state stability and particle agglomeration tendency.

## Materials and Methods

### Chemicals

D-(+)-Trehalose dihydrate (TRE-h) was purchased from Sigma-Aldrich (Lot #SLBR1467V, United States). For spray drying, TRE-h was dissolved in mixtures of deionised (DI)-water and ethanol with varying solvent ratio of 100:0 v/v, 99:1 v/v and 50:50 v/v. Solutions with TRE-concentrations of 30 mg/mL were prepared freshly before each experiment (SPT1 - SPT6). Further details on the prepared TRE solutions are listed in Table SI (ESI).

Synthesized and freeze-dried GLUC was sourced from Bachem (Lot #1056459, Switzerland). Solutions of GLUC with a concentration of 5 mg/mL (SPG1- SPG4) were freshly prepared for each experiment with 0.05 N hydrochloric acid:ethanol ratios of 100:0 v/v, 99:1 v/v and 50:50 v/v. A 1 vol% ethanol solvent ratio aimed to identify potential inter-molecular effects for the peptide stabilisation during particle formation. In comparison, a 50 vol% ethanol solvent ratio is expected to have an additional impact on process conditions including droplet atomisation and evaporation kinetics. For the GLUC-TRE formulation, 5 mg/mL of GLUC and 30 mg/mL of TRE were dissolved in 0.05 N hydrochloric acid (SPG5 F). The high mass ratio of TRE was intended to increase the final particle size during spray drying to avoid extensive fine production and provide a sufficient amorphous matrix for GLUC stabilisation. Further details on the prepared GLUC solutions are listed in Table SI (ESI). General aspects related to the use of TRE and GLUC during spray drying are included in Section SI (ESI).

### Single Droplet Drying Experiments

#### Multi-Emitter Single-Axis Acoustic Levitator

**Fig. 2:**
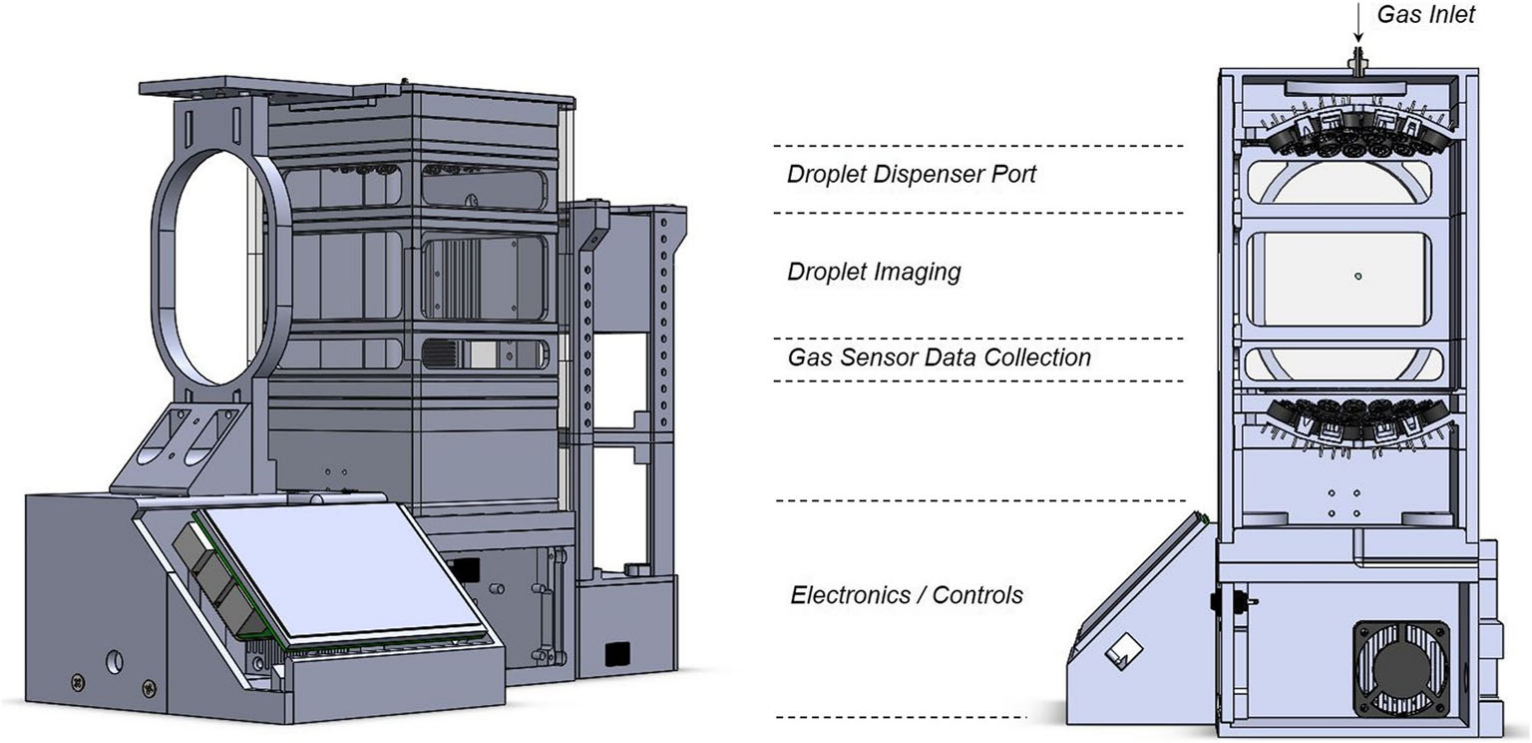
Multi-emitter Single-axis Acoustic Levitator (MSAL) used to investigate particle formation of peptide-based systems from single droplet drying experiments.

SDD experiments were performed with a Multi-emitter Single-axis Acoustic Levitator (MSAL). The MSAL was developed based on a published levitation platform [41] which was re-designed to function as an integrated characterisation platform for SDD experiments. This includes image acquisition/analysis capabilities and the implementation of a gas sensor to measure local temperature, humidity and pressure levels. The MSAL gas inlet was connected to a system from Okalab (Italy) providing a controlled dry nitrogen flow of 0.8 L/min, which was diffused over the back of the upper transducer plate. A schematic of the experimental setup is shown in Fig. 2. The droplet evaporation and solidification was recorded with a Fastcam SA1.1 high speed camera (Photron, Japan). The relative humidity, ambient temperature and absolute pressure were constantly monitored and recorded using a pre-calibrated BME280 environmental sensor (Bosch Sensortec GmbH, Germany). The relative humidity in all experiments was less than 3.5%RH with an average gas temperature in the enclosure of 34.66 ± 0.51°C. Solutions of TRE (*c*_0*,*TRE_ = 30 mg/mL), GLUC (*c*_0,__GLUC_ = 5 mg/mL) and a GLUC-TRE formulation (*c*_0*,*TRE_ = 30 mg/mL, *c*_0*,*GLUC_ = 5 mg/mL) were prepared for SDD experiments. Droplets with a volume of 5.05 ± 1.63 μl were manually suspended within the central pressure node using a Re- search plus 20 μl micropipette (Eppendorf, Germany). Image data of the SDD experiments were processed and analysed to track the droplet drying stages and determine the lock point (LP) [21]. The image analysis routine has been described previously [21]. Briefly, image processing steps included the use of an edge-preserving image filter, automatic thresholding and despeckling routines to create a binary image mask of the droplet. An ellipse with equal second order central image moments was fitted to the droplet mask in order to extract information on the major and minor axis. Both were used to calculate the droplet surface and volume assuming the shape of an oblate spheroid during droplet drying.

#### Micro-X-Ray Tomography

The structure of the dried particles was investigated using micro-X-ray tomography. The particles were scanned with a Skyscanner 2211 X-ray tomograph (NanoCT, Bruker, Kontich, Belgium) in a cone-beam arrangement. The samples were scanned with an image pixel size of 0.8 μm, frame averaging of 8 and a rotation step size of 0.2°. The X-ray acceleration voltage was 40 keV. A reference scan was collected at the end of each run to enable post-alignment and therefore compensate for potential shifts during the scan. Image reconstruction included beam hardening corrections and ring artefact reduction which were performed using NRecon with InstaRecon (version 1.7.1.6, Bruker, Kontich, Belgium). The image stacks were visualised with CTVox (version 3.2.0, Bruker, Kontich, Belgium). Image processing and analysis steps are described in details in a previous publication [21] and allow a quantitative assessment of the particle solid phase (V), of the particle region-of-interest (V_ROI_, which includes internal particle porosity) as well as of a 3D convex-hull of V_ROI_ (V_CH_) to evaluate particle convexity linked to surface buckling. The material’s true density (*ρ*_XRT_ = *m*_SDD_ */*V_V_) was estimated combining information of the solute mass in the droplet during SDD experiment (*m*_SDD_ = *c*_0,SDD_·*V*_0,__SDD_) with its final particle solid phase volume (V_V_) quantified using micro-XRT. The final particle morphology and internal micro-structure were compared after extracting 3D micro-XRT descriptors related to the particle sphericity (${\psi}_{\mathrm{gl},{\mathrm{V}}_{\mathrm{ROI}}}$= *π*^1*/*3^ (6 V_ROI,V_)^2*/*3^ */*V_ROI,A_) based on the V_ROI_ volume (V_ROI,V_) and the V_ROI_ surface area (V_ROI,A_), to the particle solidity (SV_ROI_ = V_V_ */*V_ROI,V_) and to the particle convexity (SV_CH_ = V_ROI,V_*/*V_CH,V_).

### Spray Drying

#### Spray-Drying Procedure

**Fig. 3:**
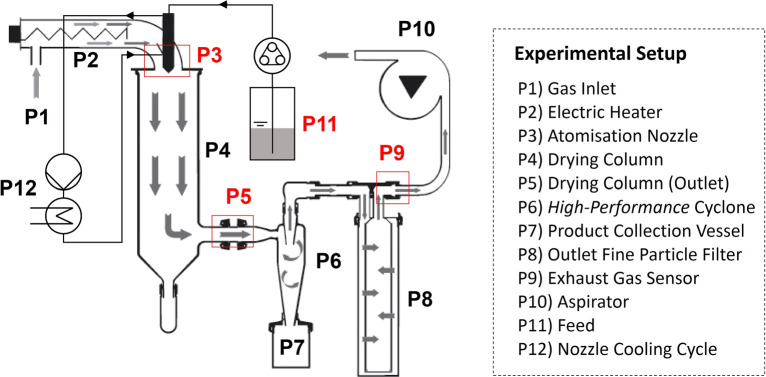
Lab-scale spray dryer (Type: B290, Büchi Labortechnik) in open loop configuration. Red markers indicate locations of process data collection. Figure adapted from Operation Manual - Mini Spray Dryer B-290 [71].

Spray drying experiments were performed using a lab-scale B-290 Mini-Spray Dryer (Büchi Labortechnik, Switzerland) in open mode configuration. The equipment set-up is shown in Fig. 3. The feed was atomised using a two-fluid nozzle with a cap orifice diameter of 0.7 mm (Fig. 3 P3). The nozzle was constantly cooled with a circulating flow from a F-25 (JULABO GmbH, Germany) set to 10°C (Fig. 3 P12). A *high performance* cyclone (Büchi Labortechnik, Switzerland, Fig. 3 P6) was employed for product recovery. The cyclone was wired with copper and grounded to reduce potential electrostatic charges building up during the solid-gas separation process, aiming to further improve product recovery, hence maximising the overall process yield. The outlet fine particle filter (Fig. 3 P8) was equipped with a PTFE membrane to allow the potential recovery of particle fines smaller than *∼* 1–2 μm.

**Fig. 4:**
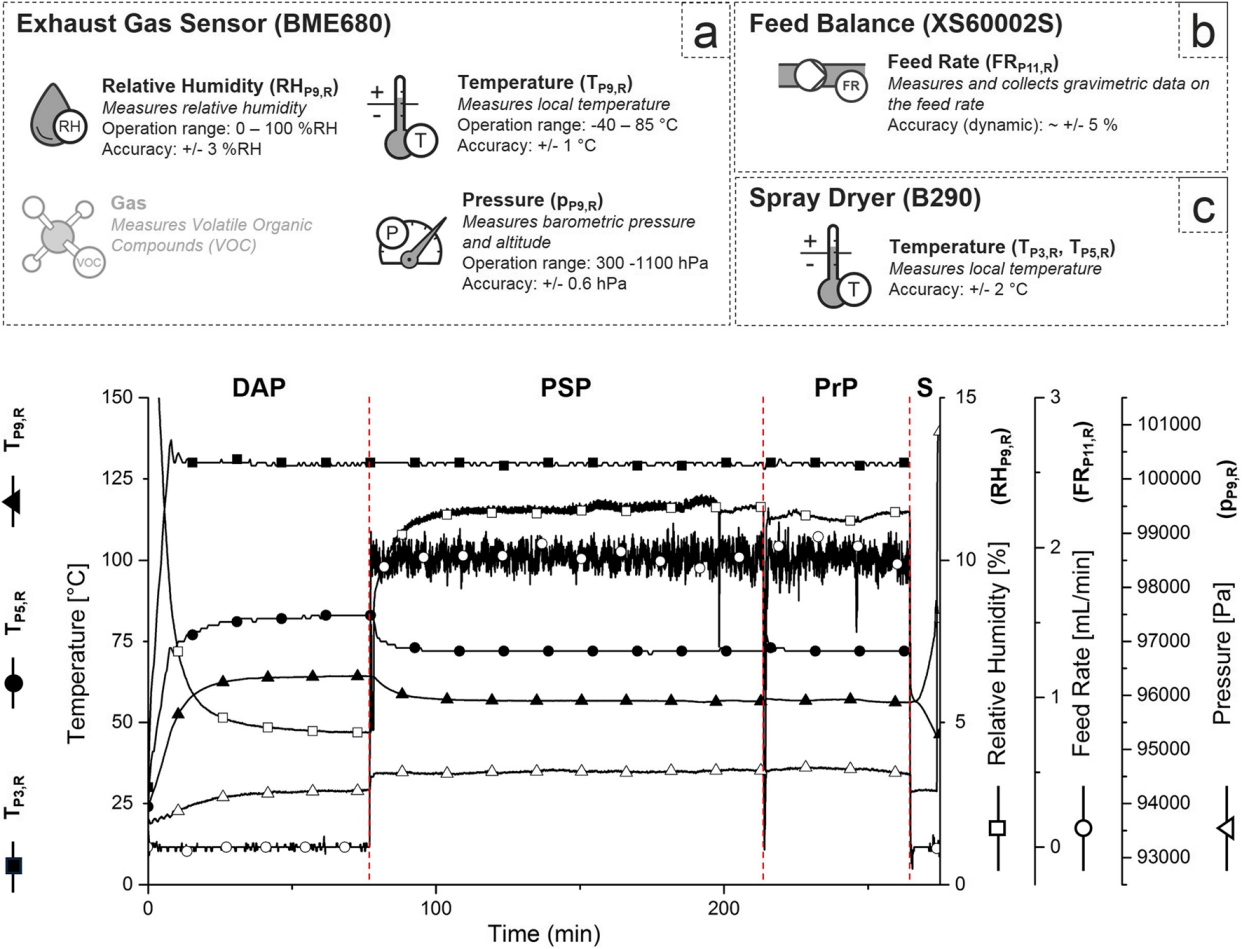
Process data of each spray drying experiment from (**a**) an implemented exhaust gas sensor, (**b**) a feed balance and (**c**) the B-290 Mini-Spray Dryer serial interface. The graph shows local temperatures (  T_P3,R_,  T_P5,S_,  T_P9,R_), relative humidity (  RH_P9,R_), local pressure (  p_P9,R_) and feed rate (  FR_P11,R_). Each process phase (DAP, PSP, PrP and S, described in Section 2.3.1) is delimited by a dashed line. Additional information on the local concentration of volatile organic compounds (VOC) in the exhaust gas were recorded but are omitted for clarity.

Each spray drying experiment can be divided in a *Dry Air* phase (DAP) to characterise the air pushed through the open loop system, a *Pure Solvent* phase (PSP) to allow system equilibration in the presence of evaporating solvent, a *Production* phase (PrP) for the spray drying of the sample solution and an instrument *Shut-down* phase (S) prior to the disassembly and cleaning of all relevant glassware. An example of all process stages and their impact on the measured process variables is shown in Fig. 4. The PSP for each experiment was >30 min until steady state conditions were reached followed by PrP of 30 min for TRE and 25 min (SPG2, SPG3, SPG5) or 50 min (SPG1, SPG4) for GLUC. PrP of GLUC was adjusted to enable sufficient material production for method development and off-line characterisation.

The process yields were calculated using Eq. 1 and are compensated against the residual moisture levels determined using TG-MS (RM_180_, see TG-MS method in Section 2.4.1).

1 $$ \mathrm{Yield}\ \left[\%\right]=\frac{\mathrm{Product}\ \left[\mathrm{mg}\right]\cdotp \left(1-{\mathrm{RM}}_{180}\left[\mathrm{wt}\%\right]\right)}{\mathrm{Solute}\ \mathrm{Concentration}\ \mathrm{Feed}\ \left[\mathrm{mg}/\mathrm{mL}\right]\cdotp \mathrm{Spray}\ \mathrm{Dried}\ \mathrm{Feed}\ \left[\mathrm{mL}\right]} $$

#### Process Data Acquisition and Integration

A gas sensor (BME680, Bosch Sensortec GmbH, Germany) was used to monitor and record temperature (TP_9,R_), relative humidity (RH_P9,R_), absolute pressure (p_P9,R_) and volatile organic solvents concentrations (VOC_P9,R_, non-calibrated) in the exhaust air. The sensor mount was designed in-house for the B-290 Mini-Spray Dryer and 3D printed using a polyjet printer (Stratasys, United States, material: Vero Black Plus RGD875, design: see Section S1. 2.1, Fig. S1 (ESI)). The integrated sensor for in-line exhaust gas analysis was installed in the gas stream after the fine particle filter (Fig. 3 P9). Additional process information from the spray-dryer was readily available via its RS232 serial interface and included selected set points (i.e. drying temperature T_P3,S_, pump speed, aspirator speed) as well as measured local temperature information at the gas inlet (T_P3,R_, Fig. 3 P3) and exiting the drying chamber (T_P5,R_, Fig. 3 P5). Information on the feed rate was recorded gravimetrically from the RS232 serial interface of a XS60002S balance (Mettler Toledo, Switzerland, Fig. 3 P11). An overview of the implemented capabilities and an example dataset is presented in Fig. 4.

#### Vacuum Drying of Spray Dried Powders

Vacuum drying was assessed as a secondary drying step for spray dried powders to further reduce residual moisture levels. Powder samples of 30–50 mg were transferred to HPLC vials and placed in a vacuum drying oven at 50°C and 20 mbar. The samples were weighed periodically until the weight reached a stable end-value which was observed after a maximum of approximately 320 h. Collected gravimetric information during vacuum drying allowed a direct comparison of its efficiency to reduce residual moisture levels as determined using TG-MS (see Section 2.4.1). Three empty HPLC vials were regularly measured at each weighing time-point as control samples. The evaluated coefficient of variation across all control measurements during the weighing process was 0.25% assuming a fixed sample mean weight of 30 mg.

### Spray Dried Powder Characterisation

#### Thermogravimetric Analysis - Mass Spectrometry (TG-MS)

TG-MS was used to quantify the residual moisture of the spray dried products and identify residual solvents. For the TG-MS analysis a TGA Q5000 (TA Instruments, United States) was connected to a ThermoStar GSD 301 T3 mass spectrometer (Pfeiffer Vacuum, Germany). Powder samples were heated to 200°C at a heating rate of 10 K/min. Mass spectra of the exhaust gas were recorded and evaluated qualitatively for changes in the ion currents linked to evaporating solvents including m/z 18 (water, H_2_O^+^) and m/z 31 or m/z 45 (ethanol, CH_3_O^+^, CH_3_CH_2_O^+^) [42].

#### Differential Scanning Calorimetry (DSC)

A *Discovery DSC* (TA Instruments, United States) was employed to evaluate product melting, dehydration/desolvation or glass transition temperatures for the produced TRE powders. For each sample, 2–8 mg of material were transferred to a crimped *Hermetic* pan (TA Instruments, United States). The pans were heated above the expected glass transition temperature (*T*_*g*_) of dry TRE at 115°C with a heating rate of 10 K/min. The *T*_*g*_ of the sample material was determined with Trios V4.0 software (TA Instruments, United States).

#### X-Ray Powder Diffraction (XRPD)

XRPD was used to test all samples for potential crystallinity. The XRPD data were collected with a D4 Endeavor (Bruker Corporation, United States) at room temperature. X-rays were generated from a copper source (Cu K*α* 35 KV *×* 50 mA). A VÅNTEC detector collected scattered light in a range between 4 and 30° (step size 0.016°, integration time 1 s). Approximately 15–25 mg of the powder samples were transferred to quartz specimen holders and levelled using a glass slide. Crystallinity in the powders was assessed qualitatively from the collected powder diffraction patterns.

#### Scanning Electron Microscopy (SEM)

Electron micrographs were collected with a Teneo SEM (ThermoFisher Scientific, United States) under low vacuum conditions of 0.4 mbar, using a large field detector, 10 kV accelerating voltage, 0.1 nA and a working distance of 10 mm. For preparation, spray dried powder samples were fixed on aluminum stubs with adhesive carbon discs. The samples were sputter coated for 90 s with 60:40 gold:palladium in a EMS575X sputter coater (Electron Microscopy Sciences) with a final coating thickness of approximately 18 nm.

#### Laser Diffraction (LD)

The particle size distribution (PSD) was evaluated with laser diffraction using a Mastersizer 2000 (Malvern Panalytical Ltd., United Kingdom) equipped with a wet dispersion unit *Hydro 2000S* (Malvern Instruments Ltd., United Kingdom). The PSD was calculated using Fraunhofer-theory (dispersant refractive index 1.38) with evaluated CVs for D_10*,*3_, D_50*,*3_ and D_90*,*3_ of 0.91%, 2.09% and 6.22% (*n* = 4), respectively. Samples were suspended in Hexane with 0.1 v/v Span80 and vortexed for 30 s prior to the initial measurement. Subsequent measurements were performed in triplicates in order to assess potential particle de-agglomeration / attrition during stirring (speed 2100 rpm). In order to further assess and compare the potential presence and strength of particle aggregates in all samples, the suspensions were exposed to ultrasound (100%) which was applied for 30 s between measurement-triplicates. The LD de-agglomeration procedure with ultrasound was repeated twice (LD0: no ultrasound, LD2: 2 *×* 30 s ultrasound). The extent of particle agglomeration was evaluated semi-quantitatively using Hartigans’ dip test (HDT) calculating the probability of unimodality (null hypothesis) [43].

#### Thioflavin T Assay

The Thioflavin T (ThT) assay enables the detection and quantification of amyloid fibril formation. For the ThT assay, powder samples were dissolved in 0.05 N hydrochloric acid and immediately transferred to a 96 multi-well plate format (MWP). The assay aimed to gather information on the potential fibrillation kinetics in the feed with increasing ethanol solvent fractions of 0.00 v/v (S1), 0.01 v/v (S2), 0.10 v/v (S3), 0.25 v/v (S4) and 0.50 v/v (S5), respectively. The ethanol was added to the aqueous GLUC solution to reach a solute concentrations of 1 mg/mL (C1), 5 mg/mL (C2) and 15 mg/mL (C3), respectively and allowed an assessment of the impact of increasing GLUC concentrations. In total, the solution in each MWP-well had a volume of 100 μL with a ThT concentration of 4 μM. The MWP was covered with a MWP-sealing tape to avoid sample evaporation. The MWP was scanned every 10 min for 24 h in a fluorescence plate reader SpectraMax i3x (Molecular Devices, Unites States) with an excitation wavelength of 450 nm and a collected emission wavelength of 480 nm. Prior to each reading cycle, the plate was shook for 3 s. The signal is directly compared to a solvent blank to detect the onset and growth kinetics during amyloid fibril formation. Additional details on the ThT assay are provided in Section S1.2.3 (ESI, ThT molecular structure Fig. S2).

#### High Performance Liquid Chromatography (HPLC)

HPLC analysis was performed using two methods for purity and potency determination which aimed to quantify impurities or degradation products and the absolute concentration of GLUC in the samples, respectively. *Impurity Method*: ACE3 C18 column (4.6 *×* 150 mm, 3.0 μm particle size) with a mobile phase A: 80/20 150 mM KH_2_PO_4_ buffer/ACN and mobile phase B: 60/40 H_2_O/ACN. *Potency Method:* Phenomenex Aeris PEPTIDE XB-C18 (3.0 *×* 150 mm, 2.6 μm particle size) with a mobile phase A: 80/20 150 mM KH_2_PO_4_ buffer/ACN and mobile phase B: 60/40 H_2_O/ACN. The 150 mM KH_2_PO_4_ buffer was adjusted with H_3_PO_4_ to pH 2.7. A pre-filtration step with a 0.22 μm PTFE filter aimed to hold back larger GLUC aggregates before diluting the solution in a 80/20 buffer/ACN stock solution to a concentration of 0.6 mg/mL. HPLC samples were collected to evaluate semi-quantitatively the potential impact of process time (Prc1 = feed sample experiment start,  Prc2 = feed sample experiment end), the aggregation and the potential degradation of GLUC after spray drying (P) and after secondary drying (VcD). HPLC measurements were performed together with standards and a control reference sample of freeze dried GLUC for each analysis run (CV 1.02%, *n* = 9). Additional details on the HPLC analysis including the mobile phase gradients are provided in Section S1.2.4 (ESI, preparation overview Fig. S3).

## Results and Discussion

### Single Droplet Drying (SDD) Experiments

**Fig. 5:**
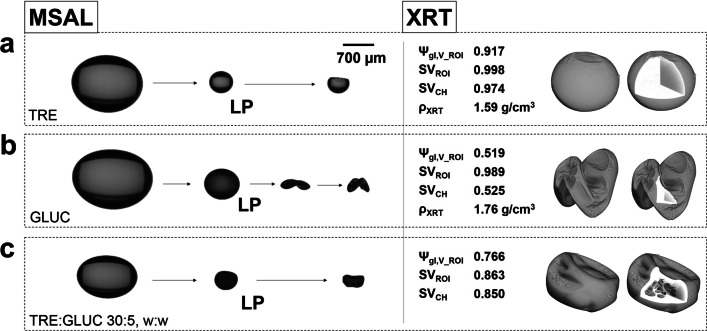
Particle morphologies of (**a**) TRE, (**b**) GLUC and (**c**) a formulation of GLUC:TRE (5:30, *w*/w) produced during SDD experiments analysed using imaging and micro-XRT. The particle models from the XRT analysis reveal their internal micro-structure and allow a quantification of particle sphericity (${\psi}_{\mathrm{gl},{\mathrm{V}}_{\mathrm{ROI}}}$), solidity (SV_ROI_), convexity (SV_CH_) and solid phase density (*ρ*_XRT_).

SDD experiments were conducted with a subsequent micro-XRT characterisation to investigate the particle formation process, drying behaviour and final particle morphologies of solution-droplets containing TRE and/or GLUC. Images of the final particle morphology from all SDD replicates (*n* = 3) are provided in Fig. S5 (ESI). Figure 5 shows the morphologies of individual particles collected from the MSAL system with quantified 3D morphological descriptors of the particle sphericity (${\psi}_{\mathrm{gl},{\mathrm{V}}_{\mathrm{ROI}}}$), the particle solidity (SV_ROI_) and the particle convexity (SV_CH_). TRE and GLUC particles from the SDD experiments exhibit distinct morphological characteristics which are consistent for each compound system. TRE particles are dense and spherical (Fig. 5a) whilst GLUC particles are highly buckled (Fig. 5b). Particles combining both, GLUC and TRE (Fig. 5c), show a reduction in the surface buckling compared to the ones of pure GLUC. Formulated GLUC particles further exhibit internal porosity that is not present in pure TRE or GLUC particles.

The particle morphologies align qualitatively with expected morphologies based on a diffusion-dominated particle formation mechanism [44]. The *Lock Point* (LP) during the droplet evaporation process is defined as the moment when the local concentration on the droplet surface (*c*_*s*_) reaches a critical value and a solid phase emerges from the solution. The Péclet number (*Pe*, Eq. 2) describes the relation between the diffusion of the solute (*D*_*s*_) and the receding droplet surface expressed through the liquid evaporation rate (*κ*). *Pe* directly impacts the surface enrichment of the solute (*E*), which is the ratio between *c*_*s*_ and the mean solute concentration in the droplet (*c*_*m*_). *E* can be estimated using Eq. 3, assuming steady-state evaporation in accordance with d2- law for *Pe* below 20 and constant *D*_*s*_ [44]. For similar liquid evaporation rates (*κ*), the difference in the diffusion coefficients (*D*_*s*_) of TRE and GLUC leads to changing levels of surface enrichment. This correlates with their radial concentration profiles between the droplet center and its receding surface. In general, a *Pe ≤* 1 leads to a flat radial concentration profile and dense, round particles as observed for TRE. *Pe »* 1 results in a non-linear increase of the radial concentration profile towards the droplet surface. For *Pe ≥* 10, this can cause early skin formation with subsequent particle buckling as observed for GLUC. Using quantified evaporation rates from the individual SDD experiments (*κ*_SDD_) and solving Eq. 3 with *c*_*s*_ = *ρ*_XRT_ (= *m*_SDD_*/*V_V_) at the point of solid/skin formation (LP), *Pe*_TRE_ and *Pe*_GLUC_ were estimated to be 6.09 *±* 0.82 (*D*_*s,*TRE_ = 0.93*·*10^*−*10^ *±* 8.51*·*10^*−*12^ m^2^/s) and 19.17 *±* 2.12 (*D*_*s,*GLUC_ = 3.16*·*10^*−*11^ *±* 4.37*·*10^*−*12^ m^2^/s), respectively. The calculated *D*_*s,*TRE_ is within the range of reported diffusion coefficients for TRE of 0.71*·*10^*−*10^ m^2^/s to 4.17*·*10^*−*10^ m^2^/s (T = 303 K) for solute concentrations between 44 and 16 wt% [45]. For TRE concentrations above 44 wt%, reported values for *D*_*s,*TRE_ rapidly decrease due to the effects of increasing solution viscosity in the proximity of the transition point between the liquid and the glassy, solid state [45]. Despite uncertainties in the estimated *Pe* values related to dynamic changes in the diffusion coefficient, the results suggest a slower diffusion of GLUC in direct comparison with TRE, which implies an expected surface enrichment of GLUC in the formulated GLUC-TRE system. Additional details of the SDD experiments including the SDD drying curves are provided in Fig. S4 (ESI).

Understanding the particle formation mechanism helps to interpret the impact of formulation and process parameters on the final particles [21]. Molecular interactions between solutes, a low solubility of individual compounds or a high surface activity can lead to significant deviations from the diffusion-dominated particle formation mechanism further emphasising the importance of small-scale droplet drying experiments [46, 47].

2 $$ Pe=\frac{\upkappa}{8\cdotp {D}_S} $$

3 $$ E=\frac{c_s}{c_m}\approx 1+\frac{Pe}{5}+\frac{Pe^2}{100}+\frac{Pe^3}{4000} $$

### Spray Drying - Platform Characterisation

The B-290 Mini-Spray Dryer platform was initially characterised to identify suitable conditions for stable process operation and support subsequent process implementation. *Dry air* (DAP) and *pure solvent* runs (PSP) were conducted with DI-water. An example dataset for assessing the response of the spray dryer at various drying temperature set-points (T_P3,S_) is provided in Fig. S6 (ESI).

#### Relative Humidity Response Surface

**Fig. 6:**
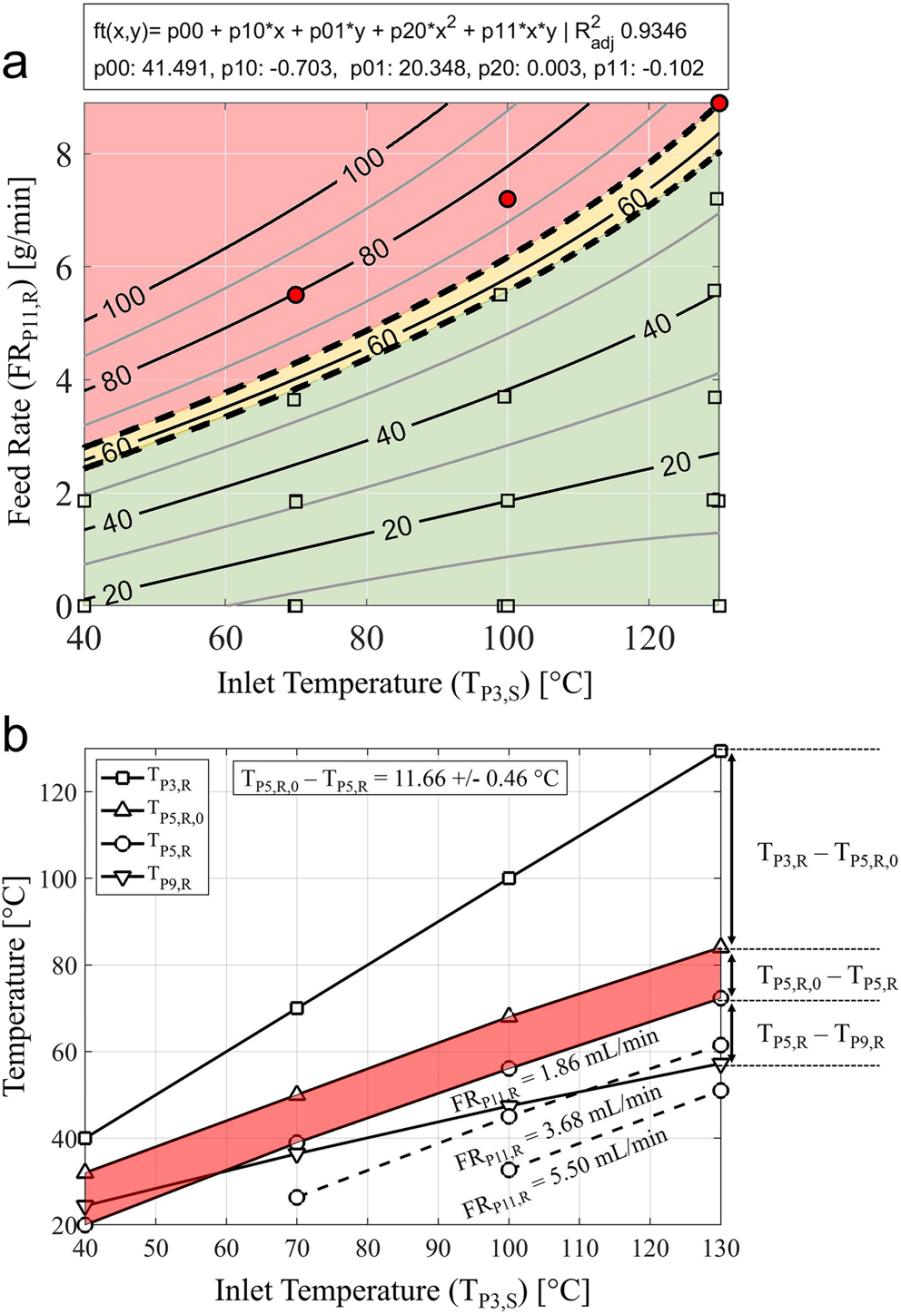
Spray dryer system characterisation: (**a**) response surface with isolines of relative humidity in the exhaust gas (RH_P9,R_) as a function of the drying temperature (T_P3,S_) and feed rate (FR_P11,R_). Process parameters with insufficient drying conditions fore pure water are marked in red. The limit for process operation ( ) was found to be between RH_P9,R_ 57.64%RH - 63.72%RH. (**b**) Basic thermal assessment for selected temperatures between 40°C and 130°C. Heat loss due to evaporative cooling (T_P5,R,0_ - T_P5,R_) was 11.66 *±* 0.46°C independent of the selected TP3,S.

The steady-state conditions for combinations of tested process variables were utilised to construct a quadratic response surface of the relative humidity in the exhaust gas (RH_P9,R_) as function of the feed rate (FR_P11,R_) and the drying temperature (T_P3,R_). The response surface is shown in Fig. 6a. PSP with insufficient drying conditions at increasing FR_P11,R_ and/or decreasing T_P3,R_ are marked in red and were excluded from the quadratic fitting approach. Insufficient drying conditions resulted in visible depositions of droplets on the wall within the top section of the drying column in direct proximity to the atomisation nozzle and/or through condensation in the cyclone. The limit for process operation (Fig. 6a, ) was defined between the iso-levels of recorded minimum and maximum RH_P9,R_ with insufficient and sufficient drying conditions, respectively. Back-projected on the response surface for RH_P9,R_, the transition zone for process operation (Fig. 6a orange) lies between 57.64%RH (max-passed) and 63.72%RH (min-failed). The relative humidity (RH_P9,R_) is not only a measure for the capacity of the drying gas to absorb additional moisture, but is also a direct indicator to evaluate the kinetics of liquid vaporization. Droplet accumulation in the drying chamber at RH_P9,R_ > 64.78%RH (Fig. 6a red) suggests that the droplet drying on this lab-scale spray dryer is mainly kinetically limited due to reduced evaporation rates at high levels of relative humidity. Information on RH_P9,R_ can be utilised to optimise the spray drying process in terms of residual moisture levels, critical particle attributes and overall process economics [18]. Higher atomization air flow rates could be used to increase the specific surface area of the droplets and further enhance liquid evaporation kinetics, which might further extend the identified zone for feasible process operation towards higher RH_P9,R_ levels. However, for this application, higher liquid atomisation was excluded to avoid risks of excessive fine production at a projected low solute concentration of less than 5 wt%.

#### Energy Balance

Temperatures in the spray dryer for selected drying temperature (T_P3,S_) between 40°C and 130°C and FR_P11,R_ of pure DI-water between 1.86 mL/min and 5.50 mL/min were measured (Fig. 6b). The temperature reduction due to evaporative cooling was directly calculated from the difference between T_P5,R,0_ during DAP (FR_P11,R_ = 0 mL/min) and T_P5,R_ during PSP, deconvoluting heat consumption for liquid vaporization and heat loss over the length of the drying column. For a FR_P11,R_ of 1.86 mL/min, the temperature decreases by 11.66 *±* 0.46°C due to evaporative cooling (Fig. 6b, marked in red) which remains almost constant over the investigated range of T_P3,S_ indicating rapid droplet evaporation in proximity of the atomisation nozzle. Temperature differences between T_P3,R_ and T_P5,R,0_ can be observed for increasing T_P3,S_ due to the heat loss in the drying column caused by thermal conduction and emission from the non-jacketed glassware. At a T_P3,S_ of 40°C and a FR_P11,R_ of 1.86 mL/min, the data shows a reversed heat-flow from the outside environment to the drying gas due to extensive evaporative cooling with a T_P5,R_ of 20.00°C below the recorded temperature of the exhaust gas (T_P9,R_ = 24.39°C).

#### Process Design Space

Approaches for the identification and selection of suitable operating conditions focus on considerations around product manufacturability. In spray drying, this is commonly related to the material’s cohesion and adhesion, referred to as stickiness, often apparent for sugar-rich materials [3, 48]. Material stickiness can lead to losses due to wall-depositions of particles within the spray dryer. The stickiness of particles containing amorphous sugars is related to the difference in the local temperature (*T*_*db*_, dry bulb temperature) from the glass transition temperature (*T*_*g*_) of the material. Reported sticky point temperatures of *T*_*db*_ *− T*_*g*_ = 15 *±* 5°C [49, 50] are not only a function of *T*_*db*_ but also the local relative humidity, altering the material’s *T*_*g*_ [51]. Moisture acts as a plasticiser lowering the glass transition temperature of the material and challenges of drying carbohydrates are commonly attributed to their high hygroscopicity [48]. Thus, a correlation between the relative humidity levels and the material’s moisture uptake is needed to predict product stickiness within the spray dryer. Literature data for water sorption isotherms of amorphous TRE are shown in Fig. S7 (ESI). The glass transition temperature of amorphous TRE (mass fraction *w*_TRE_) with changing residual moisture levels (mass fraction *w*_*w*_*)* can be estimated using the Gordon-Taylor eq. (GT, Eq. 4) [52].

4 $$ {T}_g=\frac{w_{\mathrm{TRE}}\cdot {T}_{g,\mathrm{TRE}}+K\cdot {w}_w\cdot {T}_{g,w}}{w_{\mathrm{TRE}}+K\cdot {w}_w} $$

The GT-fit for the binary TRE-water system using Eq. 4 with reported *T*_*g*_ values of water (*T*_*g,w*_ = 136 K [53]) and TRE (*T*_*g,*TRE_ = 389 K [40]) yields a GT-constant (*K*) of 6.04 (see GT-fit graph in Fig. S8 (ESI)). This *K* value lies between other published values for the TRE-water system ranging from 5.20–7.90 depending on the selected *T*_*g,w*_ and *T*_*g,*TRE_ [53–55].

**Fig. 7:**
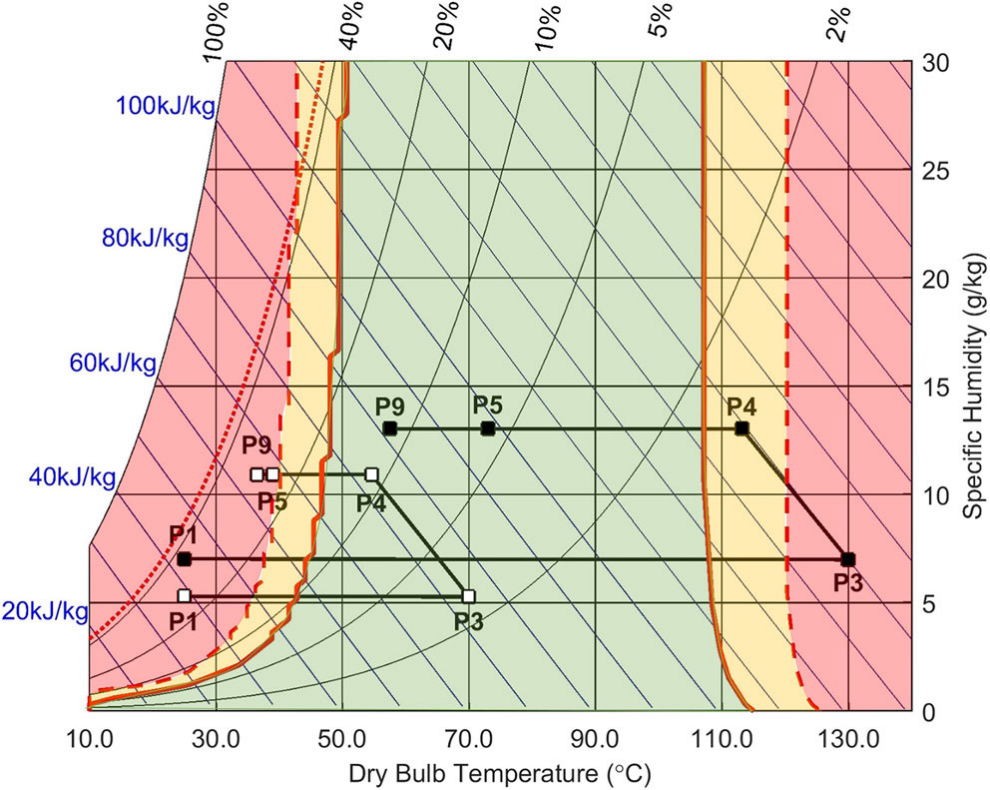
Psychrometric chart with the theoretically derived stickiness curve for the TRE-water system ( *T*_*db*_ = *T*_*g*_, *T*_*db*_ = *T*_*g*_ + 10 *K*). Risk for TRE- h nucleation above RH = 44% ( ). Experiments with T_P3,S_ 70°C ( ) and 130°C ( ) operate at distinct positions in relation to the stickiness curve. Process operation zones: (green) safe zone with *T*_*db*_ *< T*_*g*_, (yellow) transition zone with *T*_*db*_ *< T*_*g*_ + 10 *K* and (red) risk zone with *T*_*db*_ *> T*_*g*_ + 10 *K.*

Figure 7 shows the calculated stickiness curve using predicted *T*_*g*_ values of the TRE-water system within a psychrometric chart providing a practical spray dryer model to identify suitable process conditions. The graph further includes steady-state conditions within the spray dryer (P1-P9, see Fig. 3) for two selected drying temperatures, T_P3,S_ of 70°C (Fig. 7, ) and 130°C (Fig. 7, ), respectively. The temperature in P4 was estimated considering only the evaporative cooling effect whilst additional heat loss over the drying chamber wall occurs between P4 and P5 (slower heat conduction as discussed in *Energy balance*). The specific humidity in P3 - P5 was derived from measurements of the ambient air (P1) and the exhaust gas (P9). Details are provided in Section S1.3 (ESI). The process model suggests a distinct performance under each drying temperature (T_P3,S_). T_P3,S_ of 70°C passes over the material’s stickiness curve ( *T*_*db*_ = *T*_*g*_ + 10 *K)* into the process risk zone (red). This can lead to material losses at T_P3,S_ of 70°C between P5 - P9 during the particle separation process in the cyclone where there is high propensity of particle-wall interactions. T_P3,S_ of 130°C indicates stable operation with process conditions at P5 well situated in the process safe zone (green, *T*_*db*_ < *T*_*g*_). A lower T_P3,S_ of 40°C operates at relative humidity levels up to 52%RH (P9, data not shown), which are reported to carry risks for dihydrate TRE crystal formation [56, 57]. Ethanol has a *T*_*g*_ at 97 K [58] and hence its effect as a plasticiser at equal moisture levels is expected to be even more significant in direct comparison to water.

### Spray Drying - Process Implementation

Following the SDD experiments and the characterisation of the spray drying platform, the process implementation focuses on (1) the use of TRE as a potential excipient for peptide formulations to validate the process model (Section 3.3.1), (2) the translation of identified process conditions for the isolation of GLUC via spray drying (Section 3.3.2) and (3) the assessment of vacuum drying as a potential secondary, post-process drying step for spray dried powders of TRE and GLUC (Section 3.3.3).

#### Spray Drying - Trehalose

TRE was selected as a potential excipient for formulated GLUC-based systems. Initial experiments focused on an assessment of the manufacturability of TRE powders at two inlet temperature levels (T_P3,S_ low - 70°C, high - 130°C) and three ethanol solvent ratios (0 vol%, 1 vol% and 50 vol%). An overview of the results for all TRE spray drying experiments is provided in Table 1. Additional sensor data for all spray drying experiments is included in Table S2 (ESI).

**Table 1:**
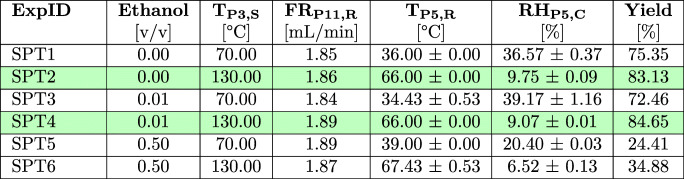
Results for the Performed TRE Spray Drying Experiments. Key Factors for a Process Evaluation were the Measured Process Conditions and Product Yields. Experiments with High Product Yields are Highlighted in Green

The achieved process yields exhibit large variations between experiments using feed solutions prepared with and without ethanol and with changing drying temperatures (T_P3,S_). Low yields in spray drying are commonly caused by (I) insufficient drying conditions leading to droplet depositions on the walls of the drying column, (II) operating above the material’s *T*_*g*_ causing high wall depositions in the drying column and cyclone [4, 18, 48] or (III) discharge of fines with the exhaust gas due to the specific cyclone performance characteristics during the solid-gas separation [4]. Droplet deposition in the drying column was not observed. A qualitative inspection of the glassware at the end of each experiment, however, suggests significant differences in local fouling for changing process conditions. Thin homogeneous films inside the drying column (Fig. 3 P4) were observed for elevated drying temperatures of T_P3,S_ 130°C, which might indicate the deposition of highly viscous, sticky particles adhering despite successful particle formation. More severe wall depositions can be observed in the cyclone (Fig. 3 P6) at reduced T_P3,S_ and especially with ethanol feed-solvent ratios of 50 vol%. As an example, Fig. S9 (ESI) provides a direct comparison of the local fouling in the cyclone at the end of the experiments SPT2 (ethanol 0 vol%) and SPT6 (ethanol 50 vol%) at T_P3,S_ 130°C. The cyclone has an estimated cut-off size ($\overline{d}$_50_) of 0.94 μm, which was calculated for the spray dryer with its specific system configuration used in these experiments (details provided in Section S1.3 (ESI)). Particles down to this size are expected to move outwards in the cyclone’s vortex and be separated. Potential fines produced during the spray drying process which are not separated from the gaseous stream in the cyclone are retained in the fine particle filter (Fig. 3 P8). An extensive deposition of fines in the filter is expected to increase the cross-filter resistance and therefore the trans-membrane pressure difference (∆p_P9_ = p_P9,R,t = 0_ *−* p_P9,R,t_). The measured values however show no significant changes in the local pressure level (∆p_P9,max_ *<* 5%), suggesting that most solids were successfully separated from the gaseous stream using the *high performance* cyclone (data listed in Table S3, ESI).

#### Spray Drying - Glucagon

The data presented in Section 3.2 and Section 3.3.1 were used as the basis for the implementation of a spray drying process for GLUC, aiming at pure compound isolation (SPG1 - SPG4) as well as the drying of GLUC formulations with TRE (SPG5 (F)). The results for spray drying experiments are shown in Table 2. Additional sensor data for all spray drying experiments is included in Table S2 (ESI).

**Table 2:**
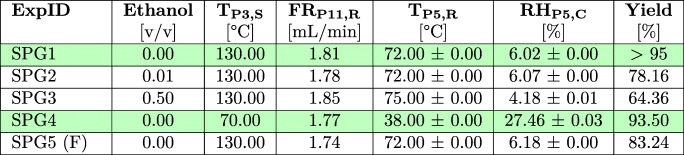
Results for the Performed GLUC Spray Drying Experiments. Key Factors for a Process Evaluation were the Measured Process Conditions and Achieved Product Yields. Experiments with High Product Yields are Highlighted in Green

The highest process yields were achieved for purely aqueous GLUC solutions with only a slight decrease of the product yield at reduced T_P3,S_, indicating that a T_P3,S_ of 70°C can be used for pure GLUC isolation as an alternative to a T_P3,S_ of 130°C to minimise any potential risk for thermal stress on the peptide material. Similar to the observations for TRE, the results demonstrate a significant impact of increasing ethanol ratios in the feed composition on the overall process yields. These are reduced by up to 32% for SPG2 (1 vol% ethanol) and SPG3 (50 vol% ethanol). In SPG5, TRE was added as an excipient to the feed solution aiming to protect the peptide against denaturation and aggregation during drying [59, 60] and improve the overall particle morphology as indicated in the SDD experiments. SPG5 exhibited a yield of 83.24%, which correlates well with yields for spray dried pure TRE of 83.13% (SPT2). The inspection of the glassware showed no significant wall-depositions in the cyclone suggesting instead an increased material loss through particle adhesion in the drying column as observed for SPT2. This indicates the dominant character of TRE for process implementation and further illustrates manufacturability risks for formulations with carbohydrates as discussed in Section 3.3.1.

#### Secondary Drying of Spray Dried Powders

**Fig. 8:**
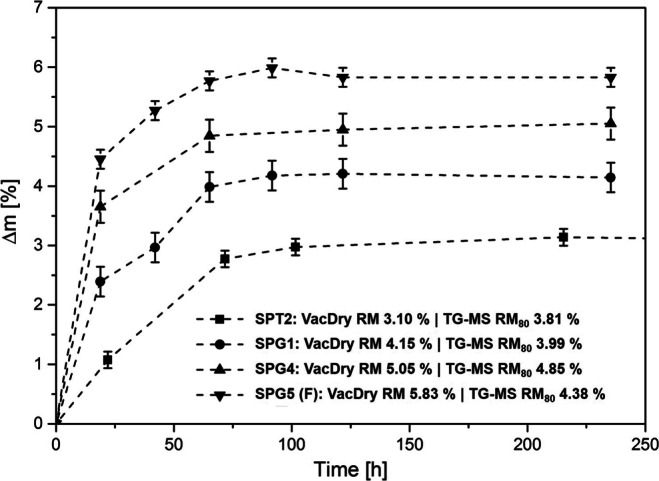
Relative mass changes (∆*m*) of spray dried TRE and GLUC samples utilising vacuum drying as a secondary, post-drying step. More than 75 wt% of the residual moisture is removed by vacuum drying at 50°C. Error bars indicate evaluated intrinsic weighing error.

For dried biological material, levels of residual moisture should generally be less than 3.0 wt% to ensure physical and chemical stability and immunologic potency for a prolonged product shelf life [61]. Vacuum drying was assessed as a secondary, post-process drying step for spray dried samples of TRE and GLUC. The measured weight changes for selected samples over a period of up to 250 h are shown in Fig. 8. Additional information on the total weight loss for all samples is included in Table 3. A stable end-weight was reached for all samples after approximately 100 h. For samples of pure GLUC, the data match well with the residual moisture levels as determined with TG-MS (see Section 3.4.1). Samples with TRE show larger differences to the TG-MS data indicating difficulties removing residual moisture in the product dried at changing drying temperature (T_P3,S_ = 70°C and 130°C). Vacuum drying is able to reduce unbound residual moisture levels of the spray dried TRE powders as determined with TG-MS (RM_80_) by more than 63.00 wt% (SPT4). For spray dried powders of GLUC, vacuum drying even reduces RM_80_ by more than 90.47 wt% (SPG3) and up to >95 wt% (SPG1, SPG4). Measured weight changes for the formulation of GLUC with TRE (SPG5) during vacuum drying significantly exceed RM_80_, which could be the result of kinetic constraints for moisture desorption during the TG-MS measurement or the desolvation of additional moisture under vacuum. The results demonstrate that vacuum drying could be utilised on lab-scale as a secondary drying step for spray dried powders produced during early process development, but is most effective for pure GLUC peptide solids. Due to the long drying times, additional process optimisation would be required to translate this approach to pilot or production scale.

**Table 3:** Residual Moisture of Spray Dried TRE and GLUC Samples Determined Using TG-MS and Vacuum Drying (VacDry). RM_80_, RM_110_ and RM_180_ Refer to the Measured Relative Mass Changes during the TG-MS Analysis at 80°C, 110°C and 180°C, respectively. TRE Powders Include Additional Information on the Measured Glass Transition Temperature (*T*_*g*_). * Detected Endotherm at *T*_*d*_ (97°C)

ExpID	TG-MS [wt%]			VacDry [wt%] RM	DSC [°C] *T*_*g*_
	RM_80_	RM_110_	RM_180_		
SPT1	2.45	4.33	8.24	6.34	42.35
SPT2	3.81	4.10	4.13	3.10	60.26
SPT3	4.78	5.82	6.48	4.70	40.32 *
SPT4	3.57	3.87	3.90	2.25	65.12
SPT5	4.77	4.90	5.58	4.28	38.98
SPT6	3.80	3.99	4.49	2.61	57.16
SPG1	3.99	4.27	7.81	4.15	–
SPG2	3.77	4.07	7.82	3.55	–
SPG3	3.78	4.19	8.08	3.42	–
SPG4	4.85	5.14	8.72	5.05	–
SPG5 (F)	4.38	7.16	–	5.83	–

### Spray Drying - Product Characterisation

Beside the identification of lab-scale spray drying conditions for the successful production of TRE and GLUC powders with high process yields, the spray dried powders were characterised off-line to study the impact of manufacturing conditions on key particle properties related to storage stability and performance. The product characterisation was focused on an assessment of particle size, morphology, residual moisture content, solid state properties and particle aggregation. The peptide-based GLUC powders were further assessed in terms of potential changes in the GLUC potency caused by peptide fibrillation or degradation during feed preparation and/or the drying process.

#### Residual Moisture and Solid State Structure

TG-MS was used to quantify residual moisture levels in the spray dried samples. The results are listed in Table 3. Plots of the thermogravimetric data are provided in Fig. S10 (ESI) and Fig. S11 (ESI) for TRE and GLUC, respectively. As expected, the residual moisture levels have an inverse correlation with the selected drying temperature (T_P3,S_). For T_P3,S_ of 70°C, residual moisture levels of spray dried TRE samples reach up to 8.24 wt% (SPT1, RM_180_). In comparison, increasing T_P3,S_ to 130°C reduces the residual moisture by 49.90% to 4.13 wt% (SPT2, RM_180_). TRE acts as a moisture sink, binding water in the form of a crystalline dihydrate (TRE-h). Therefore, assuming most of the unbound residual moisture is evaporated once the sample reaches RM_80_, additional weight changes might be linked to the dehydration of TRE-h, which mostly occurs above 97°C [62]. Any additional change between RM_80_ and RM_110_ (= ∆RM_110_) might give an indication on the extent of local TRE-h formation. Based on ∆RM_110_, the theoretical maximum crystalline content varies between 1.28 wt% (SPT5) and 19.70 wt% (SPT1). Powders produced at higher T_P3,S_ of 130°C show a stronger correlation between RM_80_ and RM_110_ suggesting no/low crystallisation of TRE-h. The solid state structure was further analysed using XRPD and DSC. The collected XRPD and DSC data for all samples are provided in Fig. S12 (ESI) and Fig. S13 (ESI), respectively. Inspection of XRPD data for all TRE samples showed no significant level of crystallinity i.e. the amount of TRE-h is below the detectable limits with SPT3 being the only exception. This aligns with collected DSC data, where a characteristic endotherm linked to TRE-h dehydration was only observed for SPT3 (*T*_*d*_ = 97°C). The data suggest that in the specific case of SPT3 the high levels of residual moisture induced a partial TRE-h formation. Measured *T*_*g*_ values of the TRE samples are included in Table 3 and correlate with recorded TG-MS data with significantly reduced *T*_*g*_ values for increasing RM levels. Additional weight changes between RM_110_ and RM_180_ (= ∆RM_180_) occur above the *T*_*g*_ of amorphous TRE (*T*_*g,*TRE_ = 115°C). Residual moisture released at these temperatures may be entrapped inside particles or within larger particle aggregates. The values correlate with selected T_P3,S_ reaching a maximum of ∆RM_180_ = 3.91 wt% for SPT1 (T_P3,S_ 70°C). Overall, the data indicates a preference for drying TRE at T_P3,S_ of 130°C to minimise residual moisture levels and to reduce the risk of local TRE-h formation. Measured residual moisture and relative humidity values match well with reported literature data at equilibrium (Fig. S7, ESI) which suggests a direct correlation between the final residual moisture levels and the local relative humidity conditions in the product collection point (Fig. 3 P7). Consequently, lower feed rates and reduced relative humidity levels could decrease the residual moisture content of the spray dried material.

Residual moisture levels in spray dried GLUC powders were less affected by changes of T_P3,S_ from 70°C to 130°C. The weight loss was measured at two main conditions at ∆RM_80_ (20–80°C = RM_80_) and ∆RM_180_ (110–180°C) for a comparison between unbound and bound moisture, respectively. ∆RM_80_ correlates with T_P3,S_, where a higher drying temperature of 130°C leads to a reduction of 17.73% comparing SPG1 and SPG4, Table 3. However, ∆RM_180_ is relatively consistent for all non-formulated GLUC powders with an average value of 3.69 *±* 0.14 wt%. For spray dried GLUC powders from pure aqueous solutions (SPG1 and SPG4), ∆RM_180_ is even more consistent with 3.57 *±* 0.02 wt% and therefore, independent of T_P3,S_. This striking consistency in ∆RM_180_ for the spray dried GLUC powders regardless of the drying temperature (T_P3,S_) shows a near constant bound residual moisture content in the absence of TRE. Molecular interactions with water are essential in the folding, stability, dynamics and function of proteins [33]. For freeze dried GLUC powders, ∆RM_180_ was significantly lower (1.83 wt%). Therefore, spray drying may show beneficial stability effects over freeze drying to preserve the potency of isolated GLUC powders. For the GLUC-TRE formulation (SPG5), temperatures over 170°C lead to a significant mass loss and browning of the powder, which indicates a Millard reaction at these temperatures. Weight changes due to peptides pyrolysis (dehydration, decarboxylation and deamination) are more commonly observed at temperatures above 180–200°C [63, 64] and were not evident in pure GLUC samples for the tested temperature range up to 200°C.

#### Particle Size and Morphology

**Fig. 9:**
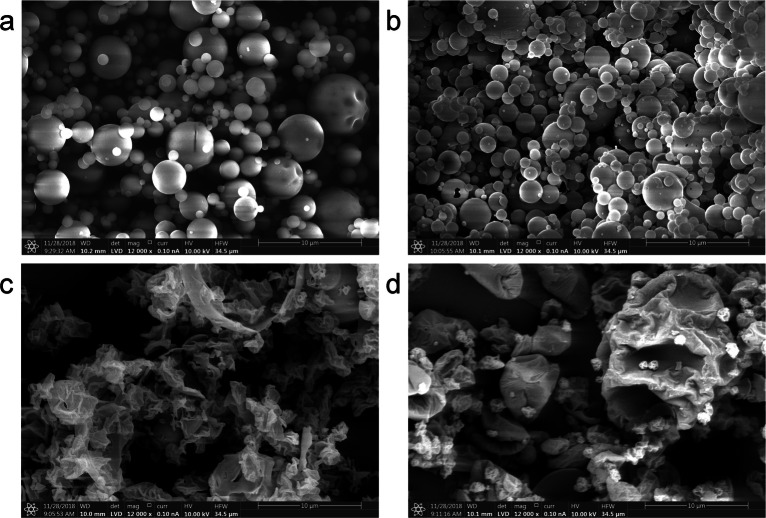
SEM micrographs of spray dried powder samples. (**a**) SPT2 (TRE, T_P3,S_ 130°C, ethanol 0.0 v/v) and (**b**) SPT6 (TRE, T_P3,S_ 130°C, ethanol 0.5 v/v), (**c**) SPG1 (GLUC, T_P3,S_ 130°C, ethanol 0.0 v/v) and (**d**) SPG5 (GLUC- TRE, 5:30 w/w, T_P3,S_ 130°C, ethanol 0.0 v/v). The images suggest an impact of ethanol on the final particle size distribution of spray dried TRE (SPT2 and SPT6). SPG1 shows an highly inflated particle morphology. The effects of surface buckling are partially reduced after the addition of TRE as demonstrated for SPG5.

Figure 9 shows SEM micrographs of selected spray dried powder samples. Additional SEM micrographs of all spray dried powder samples are provided in Fig. S14. Spray dried TRE (SPT2 and SPT6, Fig. 9a-b) exhibits a highly spherical particle morphology. The SEM images suggest a reduction of the particle size distribution (PSD) with increasing ethanol solvent ratios in the feed of up to 50 vol% (SPT6) linked to higher liquid atomisation due to the effects of reduced surface tension and/or reduced viscosity for aqueous-organic mixtures [65]. SPG1 (Fig. 9c) and SPG5 (Fig. 9d) are a direct comparison of the particle morphologies between a spray dried feed of pure GLUC and the spray dried GLUC - TRE formulation (GLUC:TRE, 5:30, w/w). The images suggest that the added TRE reduces overall particle buckling. This aligns with the observed particle morphology during the drying of the peptide formulation with TRE using SDD experiments (see Section 3.1). Here, the particle morphology was interpreted using a diffusion-dominated particle formation mechanism based on different *Pe* values for TRE and GLUC [44]. The low diffusivity of GLUC (*D*_GLUC_ *« κ → Pe »* 1) leads to early skin formation and subsequent surface buckling. This effect is further enhanced due to the rapid evaporation kinetics in the spray dryer compared to the SDD experiments (*κ*_SP_ *» κ*_SDD_). Qualitatively, the observed particle morphologies align well with reported particle morphologies for spray dried TRE [7] and larger macro-molecular peptides/proteins [66, 67].

**Fig. 10:**
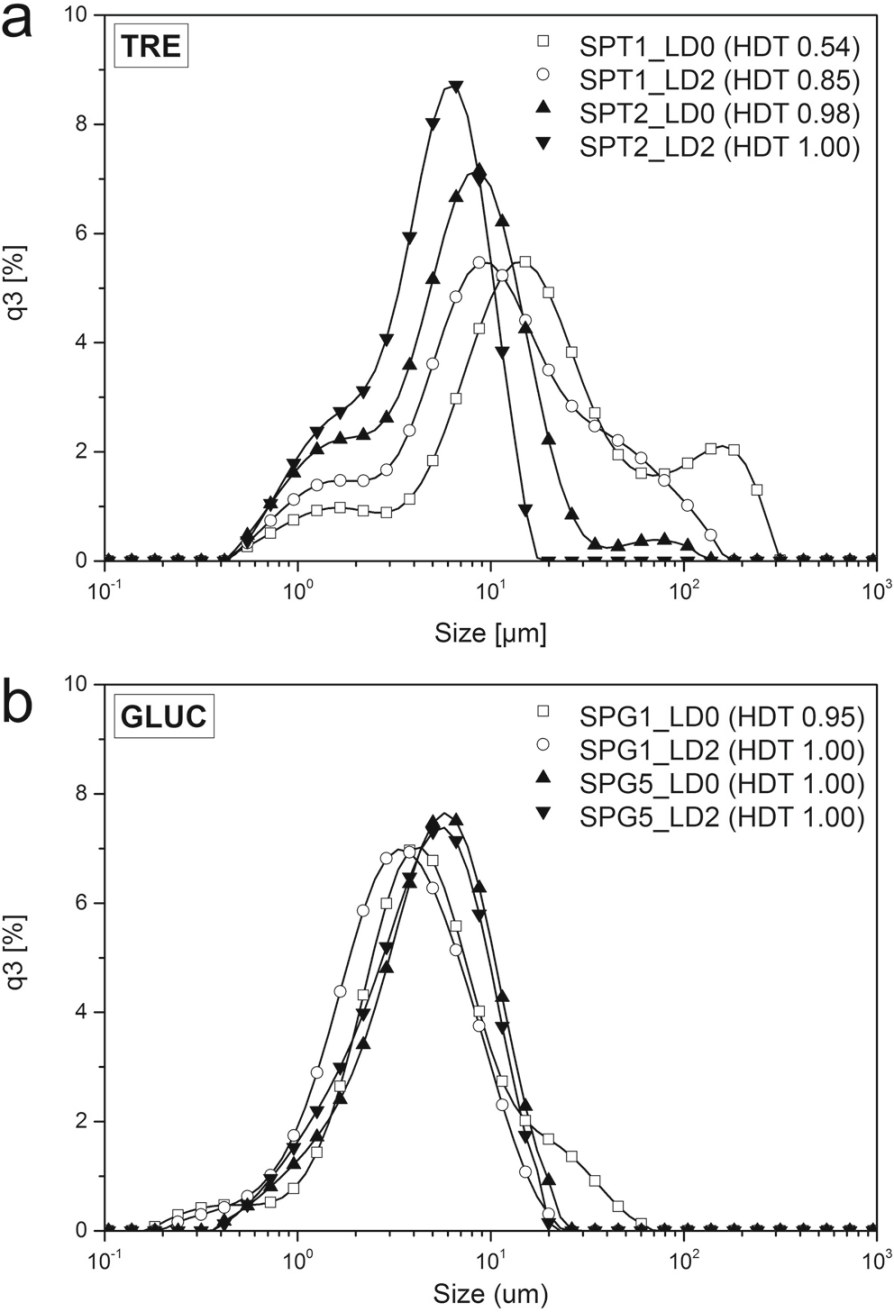
Selected PSDs of spray dried TRE and GLUC powder samples. For spray dried TRE samples, a decreased drying temperature (SPT1 T_P3,S_ 70°C, SPT2 T_P3,S_ 130°C) correlates with strong particle agglomeration creating a multi-modal PSD. Tested GLUC powders show low agglomeration characteristics in the collected PSDs even for formulations including TRE as shown for SPG5.

Quantitative information on the particle size distribution (PSD) was collected using laser diffraction (LD). The results for selected spray drying samples are displayed in Fig. 10. The D_10*,*3_, D_50*,*3_ and D_90*,*3_ of the volume-based PSD for all samples are included in Table 4. For TRE, reduced drying temperatures lead to a shift in the PSD towards larger particles with increasingly multi-modal distributions e.g. comparing SPT1 (T_P3,S_ 70°C) and SPT2 (T_P3,S_ 130°C). For pure and formulated GLUC particles, apparent changes in the PSD are mostly related to the addition of ethanol. The highest volume densities for all measured PSDs lie between 1 μm and 10 μm which aligns with the observed particle sizes using SEM (Fig. 9).

**Table 4:** Product Characterisation of Spray Dried TRE and GLUC Samples Related to Particle Size (LD2), Particle Aggregation (HDT) and GLUC Potency (HPLC). TRE Samples Exhibit a Strong Correlation between T_P3,S_ and Particle Aggregation

ExpID	LD2 [μm]			HDT		HPLC [%]			
	D_10*,*3_	D_50*,*3_	D_90*,*3_	LD0	LD2	Prc1	Prc2	P	VcD
SPT1	1.75	9.57	47.94	0.54	0.85	–	–	–	–
SPT2	1.26	4.62	9.27	0.98	1.00	–	–	–	–
SPT3	1.37	5.98	24.07	0.38	1.00	–	–	–	–
SPT4	1.20	4.40	10.16	0.81	1.00	–	–	–	–
SPT5	1.27	3.67	9.63	0.53	1.00	–	–	–	–
SPT6	1.31	3.96	9.48	0.96	1.00	–	–	–	–
SPG1	1.11	3.18	8.38	0.95	1.00	94	93	94	92
SPG2	1.25	3.75	9.87	0.78	0.99	92	92	92	97
SPG3	1.13	2.79	6.00	0.97	1.00	98	98	94	92
SPG4	1.22	3.30	7.26	0.95	1.00	92	97	93	95
SPG5 (F)	1.30	4.20	9.82	1.00	1.00	93	92	91	80

Unbound surface moisture can induce particle agglomeration and aggregation [48]. Particle agglomeration can affect powder flow and handling during downstream processing as well as potentially alter the final product performance. Spray dried TRE samples with high residual moisture exhibit highly multimodal PSDs suggesting particle agglomeration and aggregation. The extent of particle agglomeration was evaluated semi-quantitatively using Hartigans’ dip test (HDT) calculating the probability of unimodality (null hypothesis) [43]. The results are included in Table 4. As shown in Fig. 10, particle agglomeration can be partially reversed using ultrasound resulting in a shift of the PSD from larger particle sizes (> 20 μm) towards smaller particle sizes (<10 μm) and an increase of HDT (HDT_LD0_ < HDT_LD2_) after applying the LD de-agglomeration protocol (LD0: no ultrasound, LD2: 2 × 30 s ultrasound). In the case of SPT1, the data suggest that highly aggregated particles with strong bridging forces remain present even after repeated ultrasound exposures (HDT_LD2_ = 0.85). In general, the presence and strength of particle agglomerates and/or aggregates correlate with low T_P3,S_ and subsequently, high residual moisture levels. Aggregates in the TRE powders are a result of high molecular mobility leading to cohesion and unbound surface liquids which enables particle bridging and caking. Samples of spray dried GLUC did not show any evidence to suggest the presence of large amounts of particle agglomerates. Interestingly, the formulation of GLUC with TRE (SPG5) showed distinct changes in the collected PSD compared to pure TRE powders. The data suggest a significant reduction in agglomeration despite comparable residual moisture levels (RM_110_ of 7.16 wt%, Table 3) as in spray dried materials of pure TRE dried at T_P3,S_ of 70°C (RM_180_ of 5.58–8.24 wt%, Table 3), which could be explained by an enrichment of the particle surface with GLUC, inhibiting particle bridging. This is consistent with the assumption of a surface enrichment of GLUC compared to TRE due to differences in their diffusion coefficients (*D*_GLUC_ *« D*_TRE_, Section 3.1). Similar effects have been observed for other formulations containing macro-molecules such as whey proteins altering the particle surface of sugar-rich materials increasing process yields and reducing particle-to-particle and particle-to-wall stickiness [68, 69].

#### Peptide Fibrilation and Degradation

A ThT assay was employed to assess changes in the fibrillation kinetics of spray dried GLUC in direct comparison to a freeze-dried reference powder to indicate potential molecular modifications of the peptide arising from the isolation process. In accordance with previously reported observations for GLUC [70], the data show a lag time before detecting GLUC fibrillation, which is reduced with increasing GLUC concentrations. Moreover, the data suggest that the fibrillation kinetics, but less the fibrillation onset, can be further reduced with increasing ethanol solvent ratios. For ethanol solvent ratios of 50 vol%, no significant ThT fluorescence signal was detected suggesting a suppression of large GLUC fibrils with extensive *β*-sheet folding. This may suggest a potential competitive mechanism between ethanol solvation and fibril growth. A chaotropic solvational behaviour of increased ethanol solvent ratios has been described for other peptides, for instance insulin [37, 38]. From a process perspective, increased ethanol concentrations could therefore provide means to assure feed stability over prolonged manufacturing time-scales. In direct comparison to the freeze-dried material, the spray dried samples show significantly reduced levels of ThT fluorescence. Detailed results of the ThT assay for the freeze dried reference material and the spray dried samples are shown in Fig. S15 (ESI) and Fig. S16 (ESI), respectively. In applications where ethanol may promote fibrillation and has a negative effect on solution stability, pure aqueous or alternative solvent mixtures could be explored.

Spray dried GLUC powders were further characterised in terms of their post-process potency using HPLC. Together with a sample pre-filtration step to remove large peptide aggregates, HPLC analysis was performed to assess the relative level of GLUC aggregation and peptide degradation within the samples (Table 4, HPLC controls: fresh sample 92 *±* 3% potency, fibrillated *worst-case* sample 13 *±* 4% potency). The quantified GLUC concentration in the permeate (= potency) of the spray dried samples indicate no major aggregation during the manufacturing process (Table 4, Prc1 = feed sample at process start, Prc2 = feed sample at process end and P = re-dissolved spray dried product). Secondary drying in a vacuum oven (Table 4 VcD), however, leads to a reduction in the potency for SPG5 (F) suggesting that vacuum drying can be used as a secondary drying method of pure GLUC powders, but might promote GLUC aggregation and/or chemical modifications in the formulation with TRE. TRE is a non-reducing sugar and was selected to inhibit chemical reaction with the peptide in the formulation (additional details in Section S1 (ESI)). The reduced potency of SPG5 (F) after vacuum drying and evidence of strong Maillard reactions through sample browning and loss of mass above 170°C during TG-MS analysis (Fig. S11 (ESI)) suggests that some unexpected reaction occurs nonetheless in this system. Further analysis would be required to elucidate the mechanism of decomposition in TRE-GLUC formualtions. Whilst out of scope for this study, additional information on potential molecular modifications or changes in the peptide conformation during spray drying with an effect on the *in-vivo* performance would be required for a final validation of the proposed spray drying manufacturing process.

## Conclusions

Spray drying conditions were successfully identified for the efficient drying and isolation of a peptide-based GLUC formulation. Single droplet drying (SDD) experiments combined with micro-XRT analysis gave valuable insights into the particle formation process and demonstrated the impact of TRE on the final particle morphology in GLUC-TRE formulations. The final particle structure was interpreted on the basis of a diffusion-controlled particle formation mechanism, which implies an enrichment of the particle surface with GLUC.

Implemented PAT capabilities enabled an initial characterisation of the lab-scale spray drying platform, assessing independent process variables to identify feasible drying conditions for process operation. A psychrometric process model based on heat- and mass-balance considerations supported the rational selection of experiments to explore the design space for process operation. Spray drying at T_P3,S_ 130°C allowed the production of amorphous TRE powders with yields of up to 84.65% avoiding risks for partial TRE-h formation and particle agglomeration. Similarly, high yields (>95%) and comparatively low residual unbound moisture (∆RM_80_ of 3.99 wt%) was achieved for spray dried aqueous solutions of GLUC. Here, a reduced T_P3,S_ of 70°C can be considered for heat- sensitive bio-molecules. Vacuum drying was successfully used as a secondary drying step to remove >90% of unbound moisture of pure GLUC powders. Extensive GLUC fibrillation was not observed and spray dried powders retained potencies between 80% and 97% as determined with HPLC.

The experiments showed promising results using spray drying as a peptide isolation process for the rapid production of GLUC powders. The demonstrated methodologies for data capturing and analysis enable a systematic approach within a data-driven spray drying process development and implementation workflow which can be applied for the isolation of novel bio-pharmaceutical formulations on lab-scale. Linking collected off-line information to live PAT data on lab-scale could accelerate scale-up and the implementation of model predictive process control systems. The use of advanced process models, coupled with targeted material-sparing experimental platforms for data generation are key to develop, parameterise and validate new process models that are required to enable digital design, and the vision of Industry 4.0 to be realised in pharmaceutical and other process industries. Further investigations to extend the utility and predictive nature of the integrated data driven approach reported here would allow the extension of this strategy to other peptide or protein based products.

### **ACKNOWLEDGMENTS AND DISCLOSURES**

The authors would also like to thank EPSRC and the Doctoral Training Centre in Continuous Manufacturing and Crystallisation (Grant Ref: EP/K503289/1) and the EPSRC Future Continuous Manufacturing and Advanced Crystallisation Research Hub (Grant Ref: EP/P006965/1) for funding this work. The authors would like to acknowledge that parts of this work was carried out in the CMAC National Facility supported by UKRPIF (UK Research Partnership Fund) award from the Higher Education Funding Council for England (HEFCE, Grant Ref: HH13054). Data underpinning this publication are openly available from the University of Strathclyde KnowledgeBase at https://doi.org/10.15129/dcb859db-fe0d-4a56-b001-3f3d7ac6c44a.

This project was part of a pre-competitive research collaboration between the CMAC Future Manufacturing Research Hub and Eli Lilly. The authors would like to thank Ian Houson (CMAC), Rebekah Russell (CMAC) and Christopher Burcham (Eli Lilly) for their support organising this industrial collaboration. Further, the authors would like to acknowledge support in this project from Brian Pack (expertise, Eli Lilly), Mark Strege (expertise, Eli Lilly), Tim Woods (lab support, Eli Lilly), David Coates (lab support, Eli Lilly), Ryan Linder (lab support, Eli Lilly), David Remick (lab support, Eli Lilly), the SMDD Peptide Synthesis Group & Delta V Team (equipment, Eli Lilly), Robert Price (NMR, Eli Lilly), Chris Dobbins (3D printing service, Eli Lilly) and Monish Chaddha (HPLC analysis, Eurofins). Additionally, the authors would like to acknowledge Ali Anwar (CMAC) and John Robertson (CMAC), specifically for giving access to their 3D printers and helpful discussions during early prototyping of the SDD platform.

## Supplementary Information

ESM 1 (PDF 21889 kb)

## Acronyms

CV: Coefficient of variation (%)
DSC: Differential Scanning Calorimetry
GLUC: Glucagon
HPLC: High performance liquid chromatography
LD: Laser diffraction
MSAL: Multi-emitter single-axis acoustic levitator
MWP: Multi-well plate
PSD: Particle size distribution
ROI: Region-of-interest
SEM: Scanning electron microscopy
SDD: Single droplet drying
TG-MS: Thermogravimetric analysis with mass spectrometry
ThT: Thioflavin T
TRE: Trehalose
TRE-h: Trehalose dihydrate
XRPD: X-ray powder diffraction

## Symbols

*c*: Solute concentration (mg ⋅ mL^−1^)
*D*_*s*_: Solute diffusion coefficient (liquid phase) (m^2^ ⋅ s^−1^)
*E*: Surface enrichment of the solute
FR: Feed rate (S - set, R - recorded, C - calculated) (mL ⋅ min^−1^)
*m*: solute/solid mass (mg)
p: Local absolute pressure (S - set, R - recorded, C - calculated) (Pa)
p_0_: Atmospheric pressure (Pa)
*Pe*: Péclet number
RH: Relative humidity (S - set, R - recorded, C - calculated) (%RH)
RM: Residual moisture (wt%)
*T*_*d*_: Dehydration temperature (°C (K))
*T*_*db*_: Dry bulb temperature (°C (K))
*T*_*g*_: Glass transition temperature (°C (K))
T: Inlet temperature (S - set, R - recorded, C - calculated) (°C)
SV_CH_: 3D particle convexity (micro-XRT, ∈ [0*,* 1] ⊂*ℝ*^+^)
SV_ROI_: 3D particle solidity (micro-XRT, ∈ [0*,* 1] ⊂*ℝ*^+^)
V: 3D particle phase (micro-XRT)
V_CH_: 3D particle convex-hull (micro-XRT)
V_ROI_: 3D particle region-of-interest (micro-XRT)
*κ*: Evaporation rate (m^2^ ⋅ s^−1^)
ρ_XRT_: Solid phase (true) density determined using micro-XRT (g ⋅ cm^−3^)
${\psi}_{\mathrm{gl},{\mathrm{V}}_{\mathrm{ROI}}}$: Particle sphericity (micro-XRT) (∈ [0*,* 1] ⊂*ℝ*^+^)
