# Supplementary material for: Peptide Isolation via Spray Drying: Particle Formation, Process Design and Implementation for the Production of Spray Dried Glucagon
Source: Pharm Res. 2020 Dec 14;37(12):255. doi: 10.1007/s11095-020-02942-5 (PMC7736029; doi:10.1007/s11095-020-02942-5)
Supplement: Supplementary file 1 — (PDF 21889 kb) [file 11095_2020_2942_MOESM1_ESM.pdf]

Supplementary Information for "Peptide Isolation via Spray Drying: Particle Formation, Process Design and Implementation for the Production of Spray Dried Glucagon"

Frederik J. S. Doerr[a,b], Lee J. Burns[c], Becky Lee[d], Jeremy Hinds[c], Rebecca L. Davis-Harrison[c], Scott A. Frank[c], Alastair J. Florence[a,b]

[a] *EPSRC CMAC Future Manufacturing Research Hub, Technology and Innovation Centre, 99 George Street, Glasgow, G1 1RD, UK.*

[b] *Strathclyde Institute of Pharmacy & Biomedical Sciences (SIPBS), University of Strathclyde, Glasgow, G4 0RE, UK.*

[c] *Small Molecule Design and Development, Eli Lilly and Company, Indianapolis, USA.*

[d] *Eurofins Lancaster Laboratories PSS, Indianapolis, USA.*

S1.1. Chemicals

S1.1.1. Trehalose

TRE is a disaccharide comprising two glucose molecules with an alpha-alpha (1,1) glycosidic bond. It is a non-reducing sugar inhibiting reactions with amino acids in a Maillard reaction [1]. For spray drying, TRE is generally preferred over sucrose as it has a higher glass transition temperature (T_g) of 115°C compared to T_g of 74°C for sucrose [2]. TRE is commercially available as a crystalline dihydrate (TRE-h) and dehydration occurs at (T_d =) 97°C [3, 4]. Reported true density values of amorphous TRE are between 1.53 - 1.58 g/cm^3 [5, 6].

S1.1.2. Glucagon

GLUC is a single-chain polypeptide that contains 29 amino acids [7]. It is a commercial pharmaceutical hormone used against insulin-induced hypoglycemia [8]. The amino acid sequence is presented below and has an isoelectric point (IEP) of 7.21 [9]. Various groups have investigated the aggregation of GLUC in acidic and alkaline conditions identifying a complex aggregation mechanism and kinetics related to various factors including pH, concentration, temperature and hydrodynamics [10, 11, 12, 13]. The hydration of peptides or larger proteins affect their folding, stability, dynamics and function [14]. Organic solvents such as ethanol can act as chaotropic agents disrupting the hydrogen bonding network of the peptide hydration shell and furthermore, weakening hydrophobic intra-molecular interactions [15, 16, 17]. This can lead to a chaotropic solvational behaviour inhibiting ordered fibril formation of proteins, as observed for insulin at ethanol-concentrations of more than 10 wt% [18, 19].

Amino Acid Sequence: His-Ser-Gln-Gly-Thr-Phe-Thr-Ser-Asp-Tyr-Ser-Lys-Tyr-Leu-Asp-Ser-Arg-Arg-Ala-Gln-Asp-Phe-Val-Gln-Trp-Leu-Met-Asn-Thr

S1.2. Supplementary method information

S1.2.1. Spray drying - PAT implementation

A BME680 gas sensor (Bosch Sensortec GmbH, Germany) on a breakout board (Adafruit, United States) was used to monitor temperature (T), relative humidity (RH), absolute pressure (p) and volatile organic solvent concentrations (VOC) in the exhaust air of a lab-scale B-290 Mini-Spray Dryer (Büchi Labortechnik, Switzerland). The sensor mount (Fig. S1 a) was designed in-house and 3D printed using a polyjet printer (Stratasys, United States, material: Vero Black Plus RGD875). The BME680 was wired to a Arduino Nano (Arduino, Italy) using its I2C logic pins (SCK - I2C clock line, SDI - I2C data line, Fig. S1 b). The Arduino Nano sent the collected process data via its USB connection to a local computer (PC) where it was received and processed using Python (package: pyserial) to further enable data displaying and logging. An example dataset collected during the characterisation of the lab-scale B-290 Mini-Spray Dryer is shown in Fig. S6 testing platform response with decreasing drying temperature ($T_{P3,S}$) and on/off feed rate ($FR_{P11,R}$).

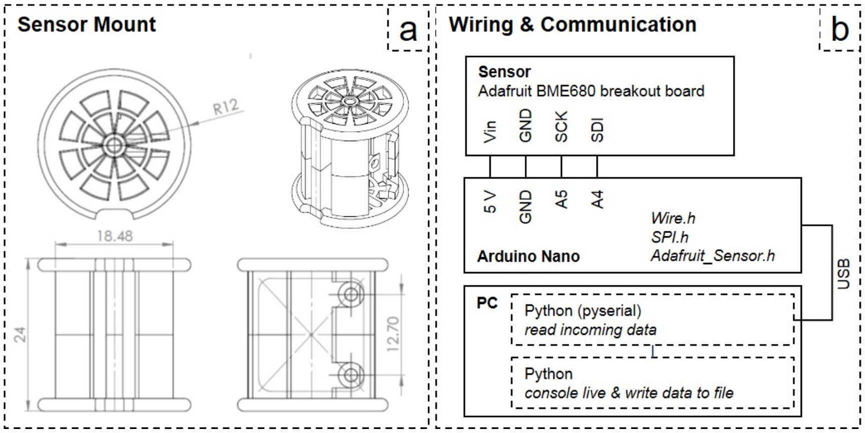

Figure S1: Implementation of the BME680 sensor recording temperature ($T_{P9,R}$), pressure ($p_{P9,R}$), relative humidity ($RH_{P9,R}$) and volatile organic solvent concentrations (VOC) of the exhaust gas at P9 (see Fig. 3). VOC data is not calibrated and was omitted from this study. a) 3D printed sensor mount with key dimensions and b) wiring of the BME680 breakout board.

S1.2.2. Spray drying - feed solution preparation

Table S1: Spray drying experiments of TRE and GLUC: Details on the prepared TRE (SPT1 - SPT6) and GLUC (SPG1 - SPG3) solutions. TRE-h = D-(+)-Trehalose dihydrate, GLUC = glucagon.

**ID**	**Compound**	**Solvent**	**Ethanol [v/v]**	***m*** [mg]	***V*** [mL]	***c*** **[mg/mL]**
Trehalose						
SPT1	TRE-h	DI-Water	0.00	3315.6	100	30.00
SPT2	TRE-h	DI-Water	0.00	3317.5	100	30.02
SPT3	TRE-h	DI-Water-Ethanol	0.01	3316.8	100	30.01
SPT4	TRE-h	DI-Water-Ethanol	0.01	3315.0	100	29.99
SPT5	TRE-h	DI-Water-Ethanol	0.50	3315.0	100	29.99
SPT6	TRE-h	DI-Water-Ethanol	0.50	3315.0	100	29.99
Glucagon						
SPG1	GLUC	0.05 M HCl	0.00	500.8	100	5.01
SPG2	GLUC	0.05 M HCl -Ethanol	0.01	250.5	50	5.01
SPG3	GLUC	0.05 M HCl -Ethanol	0.50	250.07	50	5.00
SPG4	GLUC	0.05 M HCl	0.00	501.1	100	5.01
SPG5	GLUC:TRE-h 1:6 w/w	0.05 M HCl	0.00	250.17 / 1658.3	50	5 / 30.01

S1.2.3. Product characterisation - Thioflavin T assay

The Thioflavin T (ThT) assay enables the measurement of relative changes in the fluorescence intensity of ThT upon binding to amyloid fibrils [20, 21, 22]. It can be used as part of a high-throughput method to monitor amyloid fibril formation kinetics, which allows a semi-quantitative characterisation of the aggregation kinetics for GLUC. Fibrillation is detected when the signal intensity of the fluorescence deviates from the signal baseline measured with pure solvent (blank).

The chemical structure of ThT is shown in Fig. S2. Upon binding to amyloid fibrils, the two rotational planes of both, the benzylamine and a benzathiole ring, become immobilized, which maintains the excited state of the molecule. The ThT staining efficiency depends on the fibril type. The fluorescence signal is most intense for protofilament fibrils and twisted mature fibrils [21]. Details of the ThT assay are described in Section 2.4.6.

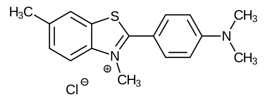

Figure S2: Molecular Structure of Thioflavin T with its two rotational planes, a benzylamine and a benzathiole ring.

S1.2.4. Product characterisation - HPLC method

HPLC analysis was conducted to detect and compare the extent of GLUC fibrillation during isolation via spray drying. As shown in Fig. S3, HPLC samples were taken from the freshly prepared feed (Prc1) as well as at the end of the spray drying experiment (Prc2) and directly diluted in the mobile phase before being filtered through a 0.22 PTFE syringe filter. The permeate was submitted for impurity detection and GLUC quantification (potency). Similarly, samples of the spray dried GLUC powders (P) and the spray dried powders after vac-

uum drying (VcD) were re-dissolved, diluted and filtered prior to the analysis with HPLC. The filtration step aimed to remove large GLUC aggregates. For a freshly prepared, *best-case* test sample and a fibrillated, *worst-case* test sample the remaining potency was 92 ± 3% and 13 ± 4%, respectively. Details of the employed HPLC methods for impurity detection and GLUC quantification (potency) are included below. The results of the impurity assay aimed to provide information on potential GLUC degradation products to further assess denaturation pathways during the isolation process, but did not show significant changes between the conditions investigated in this study and therefore, were omitted for clarity.

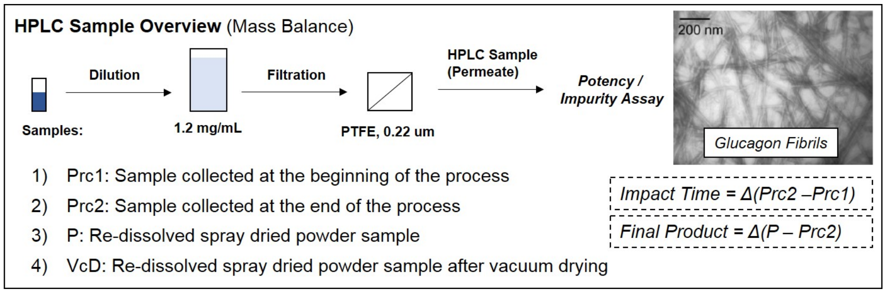

Figure S3: HPLC analysis with sample pre-filtration step was used in order to allow a semi-quantitative assessment of the peptide fibrillation. Large mature GLUC fibrils are usually highly elongated (image adapted from Pedersen et al. [21]). The samples were selected to evaluate relative changes as an indicator for feed stability (Prc1 vs Prc2) and drying (Prc2 vs P).

Details of the HPLC impurity assay as described in Section 2.4.7:

OPERATING PARAMETERS

PARAMETER	SETTING		
Flow rate	0.5 mL/min		
Gradient profile	Time (minutes)	% Mobile Phase A (80/20 Buffer/ACN)	% Mobile Phase B (60/40 H2O/ACN)
	0.0	62.0	38.0
	26.0	62.0	38.0
	30.0	12.0	88.0
	33.0	12.0	88.0
	34.0	62.0	38.0
	44.0	62.0	38.0
	*The ratio of mobile phase A and B may be adjusted to obtain a retention time of approximately 19.5 minutes.		
Auto-sampler temperature	5°C		
Detection wavelength	214 nm		
Column temperature	45°C		
Injection volume	15.0 μL		
Approximate total run time	44 minutes		
Note(s): After use, it is recommended to flush the column with 80/20 H2O/ACN to remove the high concentration phosphate buffer salt!			

89

Details of the HPLC potency assay as described in Section 2.4.7:

OPERATING PARAMETERS

PARAMETER	SETTING		
Flow rate	0.75 mL/min		
Gradient profile	Time (minutes)	% Mobile Phase A (80/20 Buffer/ACN)	% Mobile Phase B (60/40 Buffer/ACN)
	0.0*	54.0	46.0
	3.0*	54.0	46.0
	8.0	30.0	70.0
	9.0	30.0	70.0
	9.01*	54.0	46.0
	12.0*	54.0	46.0
	*The ratio of mobile phase A and B may be adjusted to obtain a retention time of approximately 5.4 minutes.		
Autosampler temperature	5°C		
Detection wavelength	214 nm		
Column temperature	45°C		
Injection volume	20.0 μL		
Approximate total run time	12 minutes		
Note(s): After use, it is recommended to flush the column with 80/20 H2O/ACN to remove the high concentration phosphate buffer salt!			

90

S1.3. Formulas

S1.3.1. Spray dryer heat- and mass-balance

Antoine equation to calculate the saturation vapour pressure at P5 ($\mathrm{p_{S,Sat,P5}}$) and P9 ($\mathrm{p_{S,Sat,P9}}$) as a function of measured local temperature (T_K [K]). For water between 0°C - 100°C: A = 10.20, B = 1730.63 and C = -39.72 [DDBST (`http://ddbonline.ddbst.com`)].

$$\mathrm{p_{S,Sat}} = 10^{A-B/(T_K+C)} \tag{S1}$$

Relative Humidity ($\mathrm{RH_{P9,R}}$) is defined as the solvent vapour pressure ($\mathrm{p_{S,P9}}$) in relation to the saturation vapour pressure ($\mathrm{p_{S,Sat,P9}}$).

$$\mathrm{RH_{P9,R}} = \frac{\mathrm{p_{S,P9}}}{\mathrm{p_{S,Sat,P9}}} \tag{S2}$$

Dalton's Rule at constant mole fractions of all inert gases for changes in the absolute pressure between local positions in the spray dryer (i = P3 - P5, $\mathrm{p_{P3}} = \mathrm{p_{P4}} = \mathrm{p_{P5}} \backsimeq 1$ atm $= 1.013 \cdot 10^5$ Pa) and P9 ($\mathrm{p_{P9,R}}$).

$$\mathrm{p_{S,i}} = \mathrm{p_{S,P9}} \frac{\mathrm{p_i}}{\mathrm{p_{P9,R}}} \tag{S3}$$

$$\mathrm{RH_{i,C}} = \mathrm{RH_{P9,R}} \cdot \frac{\mathrm{p_i}}{\mathrm{p_{P9,R}}} \cdot \frac{\mathrm{p_{S,Sat,P9}}}{\mathrm{p_{S,Sat,i}}} \tag{S4}$$

S1.3.2. Cyclone separation

The solid-gas separation in a cyclone can be described using Barth's classical model [23]. The critical cut-off size of the cyclone ($\bar{d}_{50}$, Equation S5) is a function of air viscosity (η), radial air velocity (v_{r_i}), radius of the exit duct (r_i), tangential particle velocity (v_t), and particle density (ρ_p). Additional information on the volume flow rate (Q), the internal radius of the cyclone (r_a) and the

vortex height (h) are needed to estimate v_{r_i} and v_t [24].

$$\bar{d}_{50} = \sqrt{\frac{18 \cdot \ \eta \cdot \ v_{r_i} \cdot \ r_i}{v_t^2 \cdot \ \rho_p}} \tag{S5}$$

with radial air velocity:

$$v_{r_i} = \frac{Q}{2\pi r_i h}$$

and tangential particle velocity:

$$v_t = v_{in}\sqrt{\frac{r_a}{r_i}}$$

The volume flow rate (Q) of 15.51 m^3/h was estimated according to Equation S6 using information on the specific humidity change between P3 and P5 ($\mathrm{SH_{P5\text{-}P3}}$) and the recorded feed rate ($|\mathrm{FR_{P11,R}}|$).

$$Q = \frac{|\mathrm{FR_{P11,R}}|}{\Delta\mathrm{SH_{P5\text{-}P3}}}\frac{R \cdot \ T}{p} \tag{S6}$$

with the ideal gas constant (R), local temperature (T in K) and local absolute pressure ($\mathrm{p_{P9}} \simeq 1.013 \cdot 10^5$ Pa). Values for the calculation of the cyclone's cut-off size ($\bar{d}_{50}$) to evaluate the cyclone separation:

Z_0 [m]	r_i [m]	r_a [m]	η [Pa s]	ρ_p [kg/m^3]	Q [m^3/s]	$\bar{d}_{50}$ [m]
$140 \cdot 10^{-3}$	$7 \cdot 10^{-3}$	$19.4 \cdot 10^{-3}$	$1.82 \cdot 10^{-5}$	1470	$4.3 \cdot 10^{-3}$	$9.81 \cdot 10^{-7}$

S1.4. Supplementary Experimental results

S1.4.1. Single droplet drying (SDD) experiments

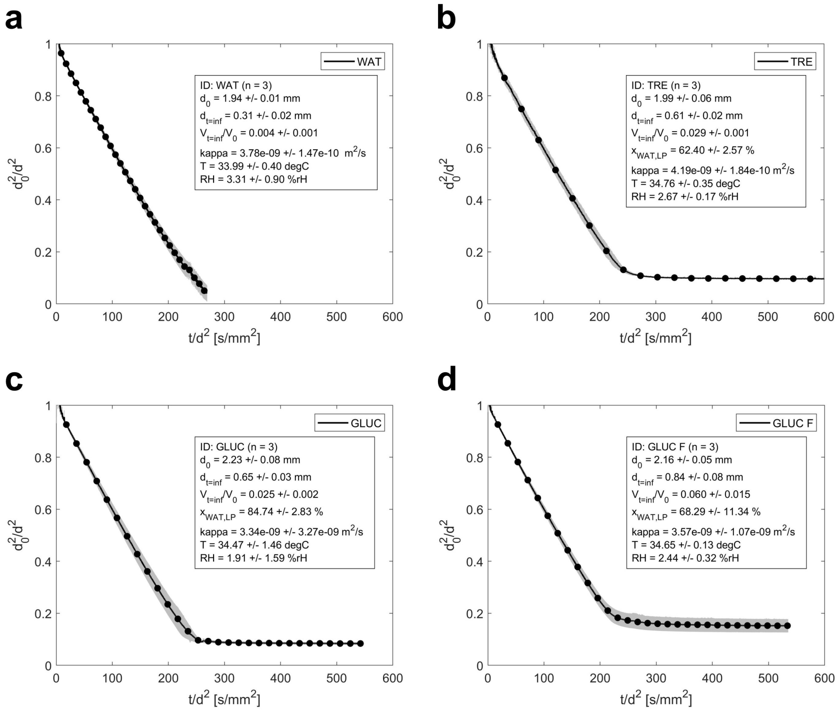

Figure S4: Drying curves of TRE, GLUC and a formulation of GLUC-TRE recorded during SDD experiments on the MSAL. (a) pure water (WAT), (b) TRE c_0 30 mg/mL, (c) GLUC c_0 5 mg/mL and (d) GLUC c_0 5 mg/mL formulation with TRE c_0 30 mg/mL. Annotations: n = number of experiments, d_0/V_0 = starting droplet diameter/volume, $d_{t\,=\,inf}/V_{t\,=\,inf}$ = final droplet or particle diameter/volume, $x_{WAT,LP}$ = WAT mass fraction at LP, kappa = evaporation rate, T = MSAL ambient temperature and RH = MSAL ambient relative humidity.

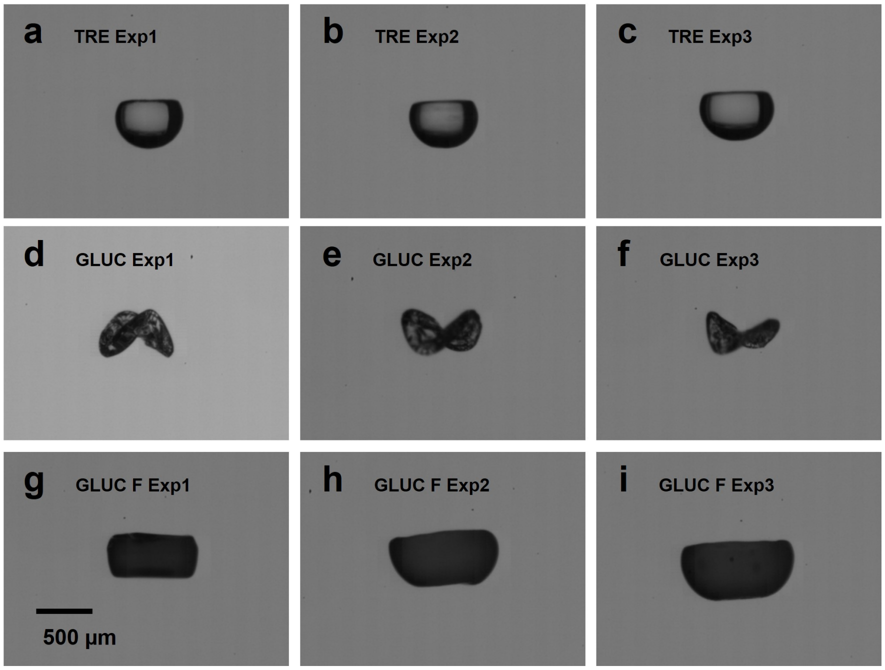

Figure S5: Images of the final particle morphology at the end of individual SDD experiments levitated in the MSAL for TRE (a-c, c_0 30 mg/mL), GLUC (d-f, c_0 5 mg/mL) and a formulation of GLUC-TRE (g-i, GLUC F with GLUC c_0 5 mg/mL and TRE c_0 30 mg/mL). The particle morphology is highly consistent across the performed SDD experiment triplicates for each compound system.

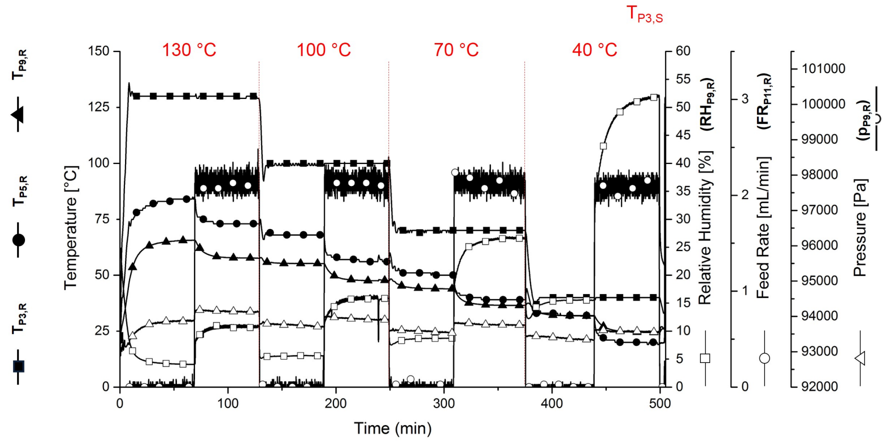

Figure S6: Example dataset for Büchi B290 spray dryer system characterisation assessing impact of selected inlet temperature ($T_{P3,S}$) and feed rate ($FR_{P11,R}$) on measured levels of temperature ($T_{P3,R}$, $T_{P5,R}$ and $T_{P9,R}$), pressure ($p_{P9,R}$) and relative humidity ($RH_{P9,R}$).

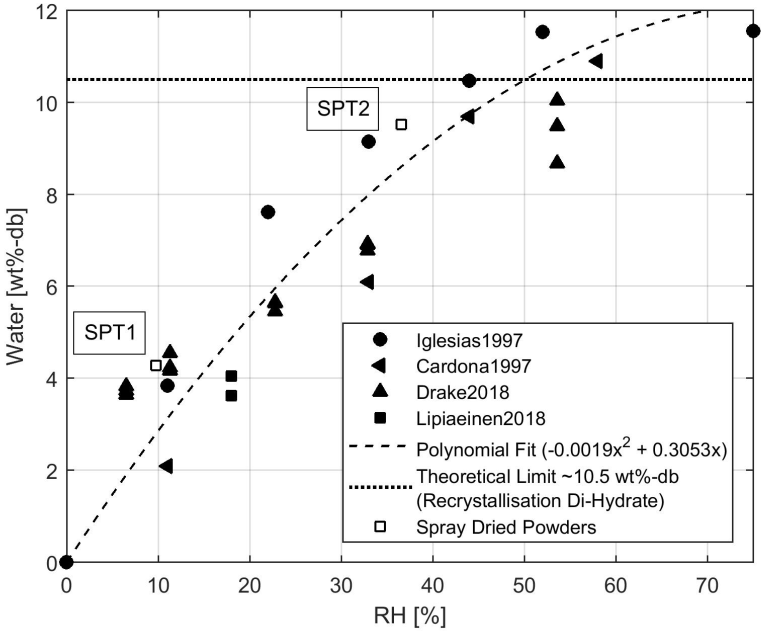

Figure S7: Water sorption isotherm of amorphous TRE at 25°C from published data [25, 26, 27, 28] and measured moisture content for experimental spray dried samples (SPT1, SPT2). The published data was used to correlate relative humidity levels ($RH_{P5,C}$) measured during the system characterisation with expected residual water content in the spray dried material. Deviations in reported T_g values might be due to a adsorption/desorption hysteresis.

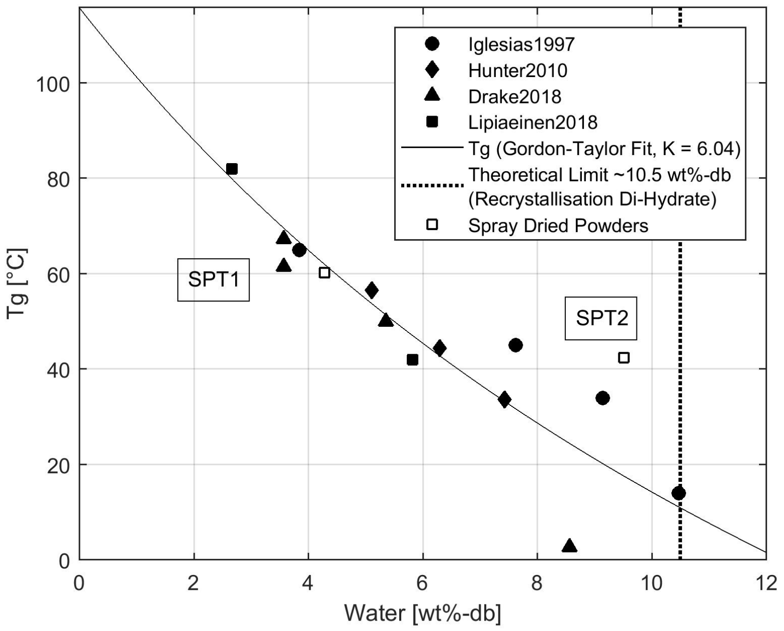

Figure S8: Reported glass transition temperature (T_g) values of TRE samples with changing moisture content [25, 29, 27, 28].

S1.4.3. Spray drying - process implementation

Table S2: Spray drying experiments of TRE and GLUC: Overview of collected process data. B290 process data was transferred through the serial interface of the B-290 Mini-Spray Dryer. BME process data were readily available from a pre-calibrated environmental sensor. $RH_{P5,C}$ was calculated assuming atmospheric pressure at P5 (see Fig. 3).

**ID**	**B290 Process Data**		**BME Process Data**			
	$T_{P3,R}$ [°C]	$T_{P5,R}$ [°C]	$RH_{P5,C}$ [%]	$T_{P9,R}$ [°C]	$RH_{P9,R}$ [%]	$p_{P9,R}$ [Pa]
Trehalose						
SPT1	70.00 ± 0.00	46.71 ± 0.49	20.54 ± 0.52	34.04 ± 0.03	36.88 ± 0.28	91677.26 ± 28.79
SPT2	129.71 ± 0.49	80.00 ± 0.00	5.35 ± 0.00	53.62 ± 0.06	15.77 ± 0.15	92273.36 ± 24.69
SPT3	70.00 ± 0.00	48.00 ± 0.00	19.09 ± 0.00	33.40 ± 0.04	37.49 ± 0.20	91112.00 ± 28.16
SPT4	130.29 ± 1.38	73.71 ± 1.80	6.51 ± 0.52	52.13 ± 0.03	15.71 ± 0.06	91636.45 ± 24.94
SPT5	70.14 ± 0.38	48.00 ± 0.00	12.77 ± 0.00	35.79 ± 0.01	22.16 ± 0.03	92451.49 ± 24.00
SPT6	129.86 ± 0.90	76.86 ± 0.69	4.43 ± 0.13	50.86 ± 0.38	12.92 ± 0.21	92501.45 ± 17.61
s-Glucagon						
SPG1	129.43 ± 0.53	83.00 ± 0.00	3.83 ± 0.00	56.20 ± 0.01	11.47 ± 0.02	94571.32 ± 10.17
SPG2	129.57 ± 0.53	83.00 ± 0.00	3.86 ± 0.00	56.39 ± 0.19	11.18 ± 0.07	92905.55 ± 10.85
SPG3	130.00 ± 0.00	83.57 ± 0.53	2.96 ± 0.06	56.32 ± 0.22	8.89 ± 0.15	94149.15 ± 10.76
SPG4	69.43 ± 0.53	49.00 ± 0.00	15.50 ± 0.00	35.81 ± 0.01	28.21 ± 0.03	92482.85 ± 6.77
SPG5 (F)	130.00 ± 0.00	83.00 ± 0.00	3.93 ± 0.00	56.70 ± 0.04	11.29 ± 0.03	92880.98 ± 8.57

Table S3: BME680 sensor data for spray drying experiments of TRE evaluating pressure differences related to changes in the cross-filter resistance due to product deposition in the B-290 Mini-Spray Dryer fine particle filter. No significant changes were detected ($\Delta p_{P9,max} < 5\%$) suggesting that most solids were successfully separated from the gaseous stream using the *high performance* cyclone. $p_{P9,R}$ data of each experiment were fitted using a linear regression (m_{LinReg} = slope, t_{LinReg} = offset).

**Experiment**	c_{Solute} [mg/mL]	**Ethanol** [v/v]	$T_{P3,S}$ [°C]	**BME** $p_{P9,R}$ **[Pa]** m_{LinReg} [Pa/min]	t_{LinReg} [Pa]	$\|\Delta p_{P9,max}\|$ [Pa]
Trehalose						
SPT1	30	0.00	70	-7.54	9.26E+04	285
SPT2	30	0.00	130	-1.60	9.25E+04	169
SPT3	30	0.01	70	-2.32	9.14E+04	145
SPT4	30	0.01	130	-1.13	9.19E+04	193
SPT5	30	0.50	70	0.16	9.24E+04	197
SPT6	30	0.50	130	-3.37	9.30E+04	243
s-Glucagon						
SPG1	5	0.00	130	-1.36	9.50E+04	141
SPG2	5	0.01	130	0.96	9.26E+04	69
SPG3	5	0.50	130	-5.50	9.53E+04	174
SPG4	5	0.00	70	-1.67	9.31E+04	187
SPG5	35	0.00	130	1.36	9.25E+04	67

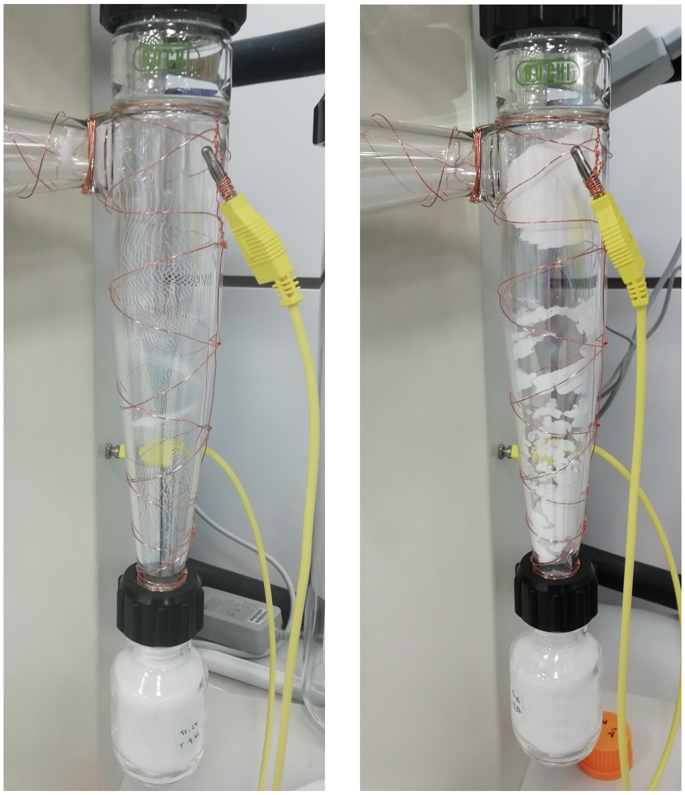

Figure S9: *High performance* cyclones after spray drying experiments (left) SPT2 ($T_{P3,S}$ 130°C, ethanol none) and (right) SPT6 ($T_{P3,S}$ 130°C, ethanol 50 vol%) showing significant differences in the local particle adhesion leading to wall-depositing of material in the cyclone.

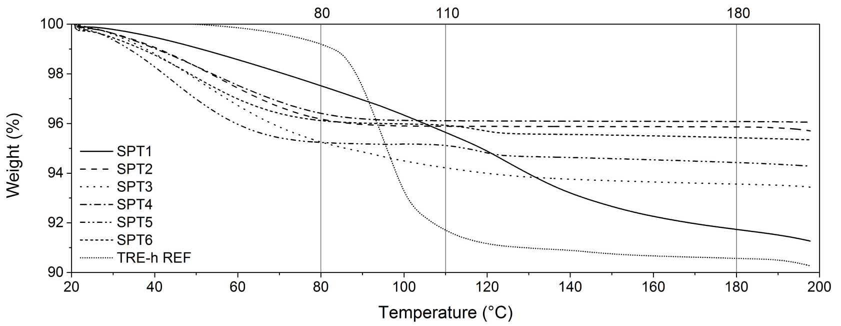

Figure S10: TG-MS data of spray dried TRE powders. The weight loss was subdivided in three steps: ΔRM_{80} (20 - 80°C) indicating residual unbound moisture, ΔRM_{110} (80 - 110°C) indicating TRE-h as observed for the TRE-h reference material and ΔRM_{180} (110 - 180°C) above $T_{g,\mathrm{TRE}}$ of 115°C.

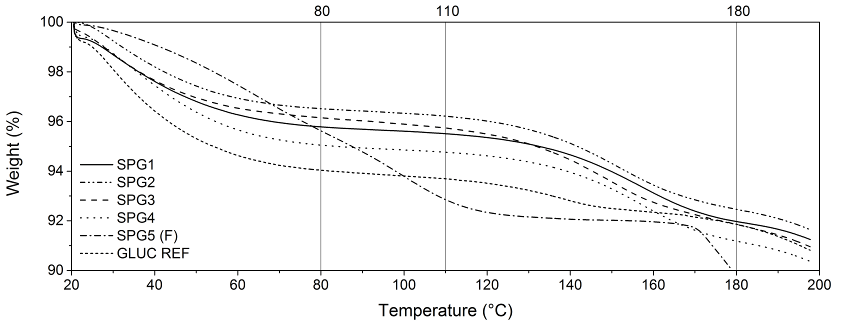

Figure S11: TG-MS data of spray dried GLUC powders. Most of the weight loss occurs in two steps between at ΔRM_{80} (20 - 80°C) and ΔRM_{180} (110 - 180°C). ΔRM_{80} relates to unbound residual moisture. In contrast, ΔRM_{180} might indicate the amount of bound residual moisture. SPG5 exhibits rapid weight losses above 170°C and extensive browning indicating a Maillard reaction at these temperatures.

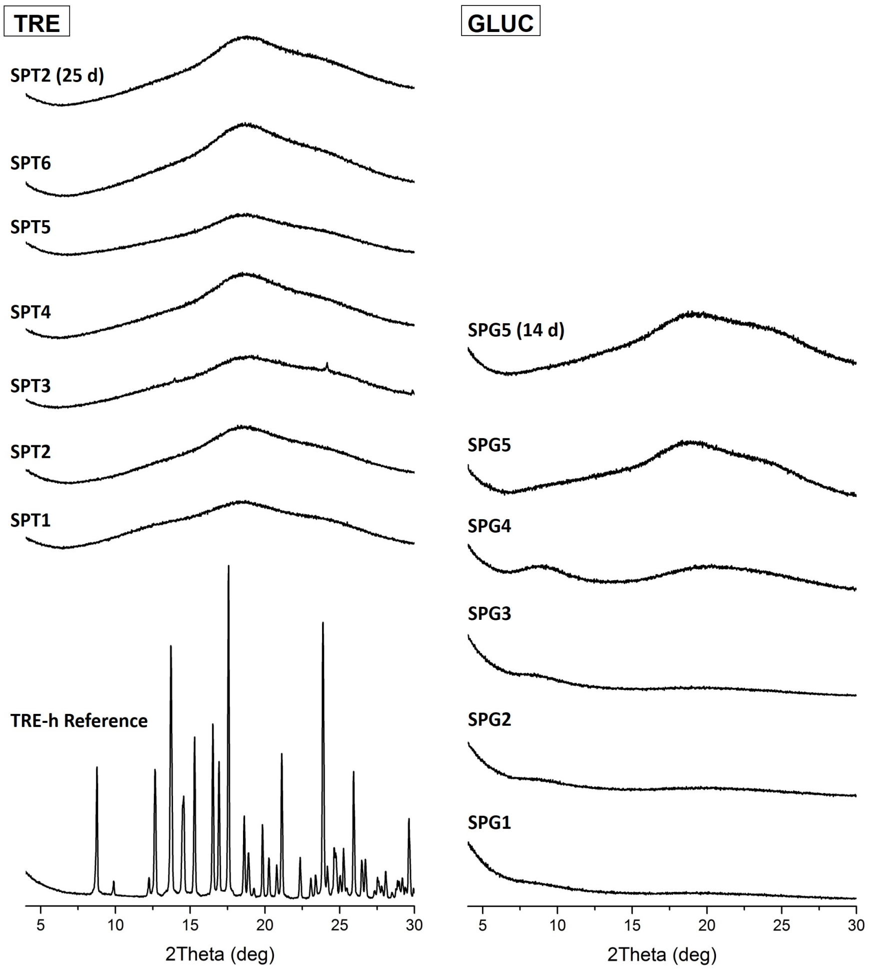

Figure S12: XRPD data of (left) spray dried TRE and (right) GLUC powders suggest an amorphous solid state structure of the produced solids below the XRPD detection limit, except for SPT3. The amorphous solid phase of the TRE matrix aims to protect the peptide against denaturation (water replacement theory [30, 31]). SPT2 and SPG5 were re-scanned after 25 days and 14 days, respectively.

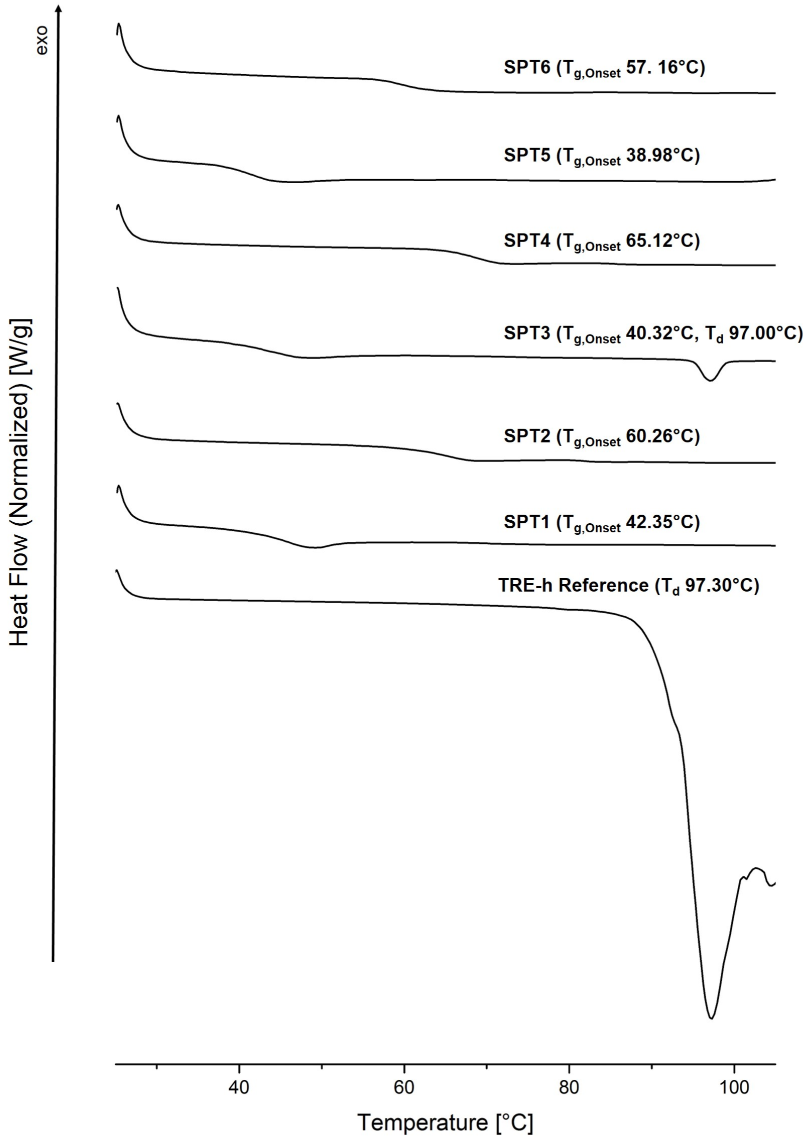

Figure S13: DSC data of spray dried TRE powders suggest an amorphous solid state structure of the produced solids, except for SPT3 with a dehydration peak (T_d) at 97.00°C. The glass transition temperatures (T_g) of the TRE powders are between 38.98°C (SPT5) and 65.12°C (SPT4).

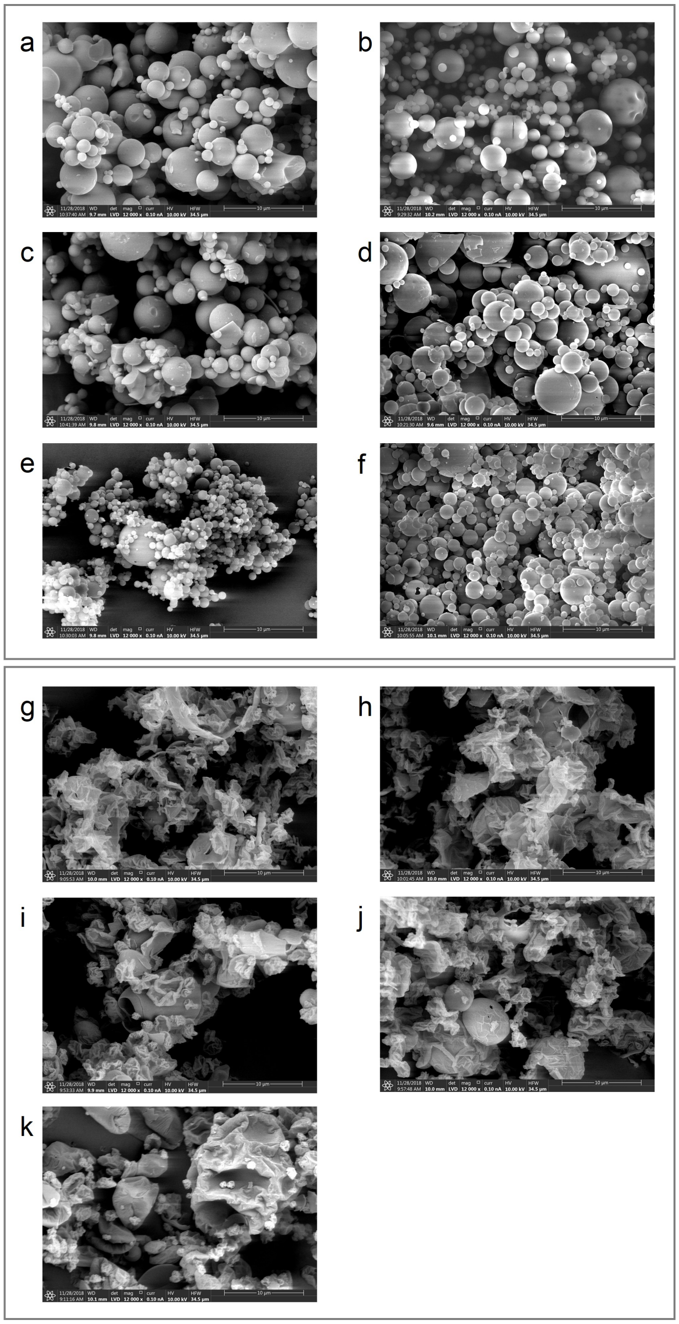

Figure S14: SEM images of spray dried TRE and GLUC samples. TRE samples: (a) SPT1, (b) SPT2, (c) SPT3, (d) SPT4, (e) SPT5, and (f) SPT6. GLUC samples: (g) SPG1, (h) SPG2, (i) SPG3, (j) SPG4, and (k) SPG5.

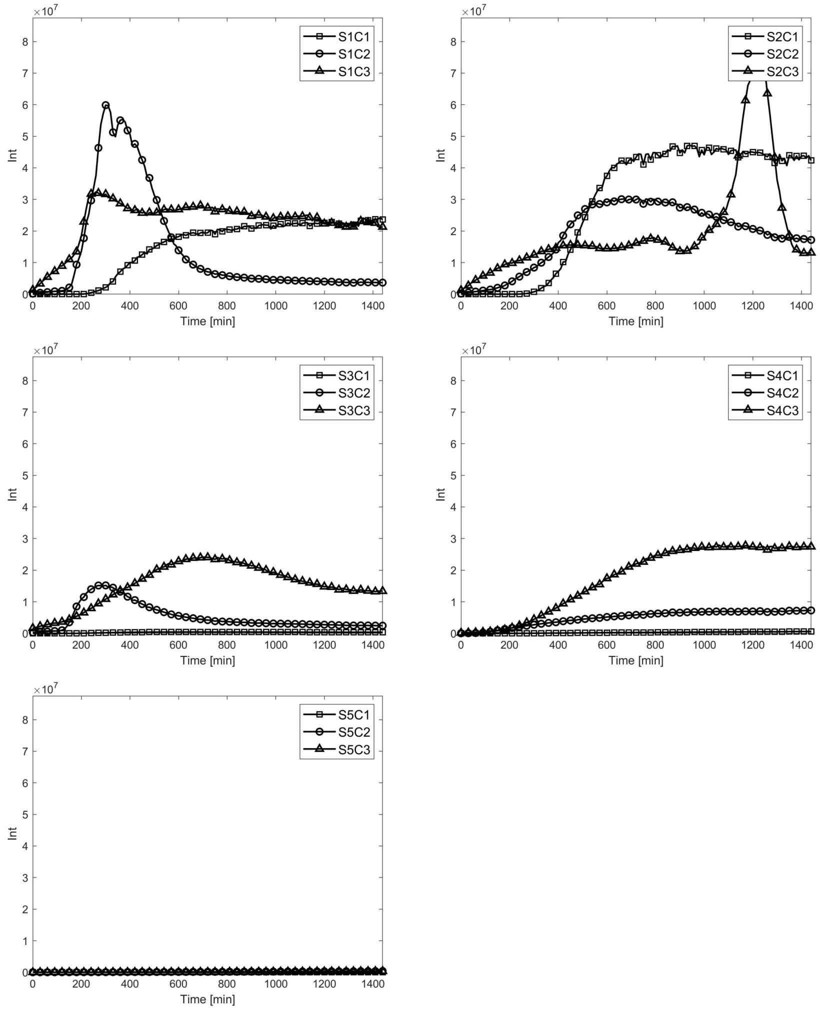

Figure S15: ThT assay for kinetic assessment of freeze-dried GLUC reference powder regarding feed stability (onset of fibrillation) and fibrils growth kinetics at changing ethanol solvent ratios (S1 = none, S2 = 1 vol%, S3 = 10 vol%, S4 = 25 vol%, S5 = 50 vol%) and changing GLUC concentrations (C1 = 1 mg/mL, C2 = 5 mg/mL, C3 = 15 mg/mL). The collected data suggest a change in the fibrillation kinetics leading to changes in the ThT fluorescence. At a ethanol solvent ratio of 50 vol%, the ThT fluorescence is completely suppressed. All values are calculated means from sample triplicates. The signal of solvent blanks was subtracted to adjust the baseline.

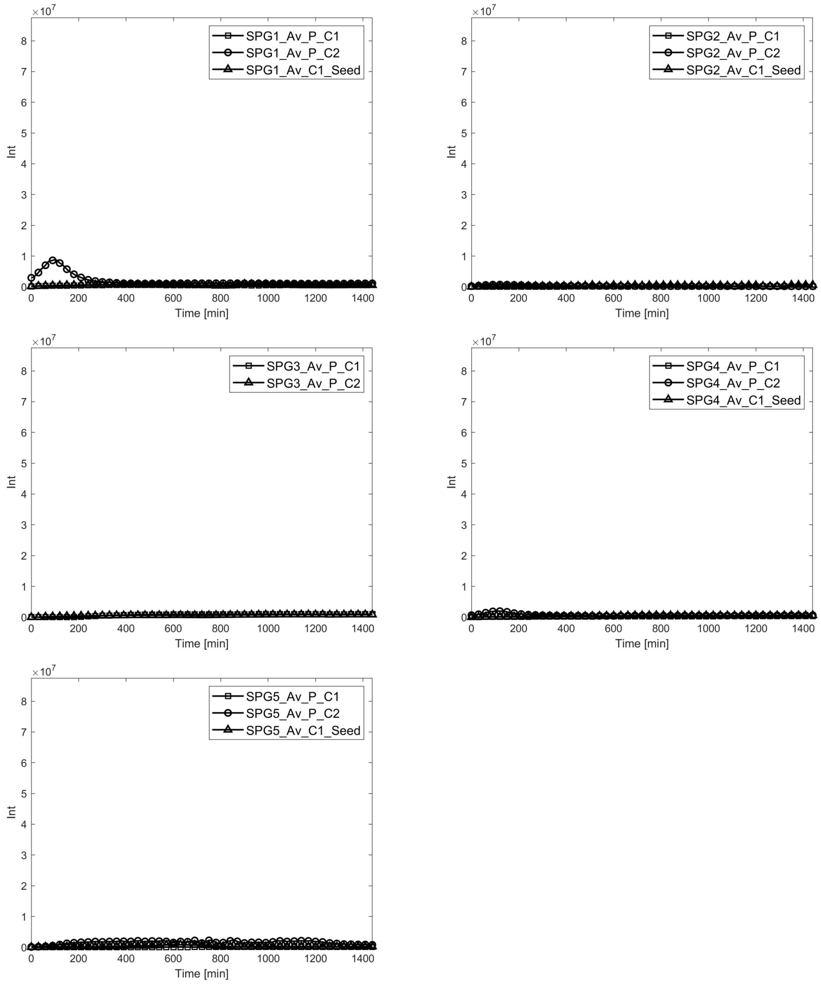

Figure S16: ThT assay of spray dried GLUC powders in 0.05M HCl at two concentrations (C1 = 1 mg/mL, C2 = 5 mg/mL) and seeded with 5 μl of a suspension of a previously aggregated GLUC sample. The data suggest that the fibrillation kinetics or the fibrillation pathway of the spray dried GLUC powders are significantly altered in direct comparison to data collected for the freeze dried reference material presented in Fig. S15. All values are calculated means from sample triplicates. The signal of solvent blanks was subtracted to adjust the baseline.

References

[1] Takanobu Higashiyama. Novel functions and applications of trehalose. *Pure and Applied Chemistry*, 74(7):1263–1269, 2002.

[2] Azita Saleki-Gerhardt and George Zografi. Non-Isothermal and Isothermal Crystallization of Sucrose from the Amorphous State. *Pharmaceutical Research*, 11(8):1166–1173, aug 1994. ISSN 1573-904X. doi: 10.1023/A:1018945117471.

[3] Fabiana Sussich, Ranieri Urbani, Francesco Princivalle, and Attilio Cesàro. Polymorphic amorphous and crystalline forms of trehalose. *Journal of the American Chemical Society*, 120(31):7893–7899, 1998. ISSN 00027863. doi: 10.1021/ja9800479.

[4] Satoshi Ohtake and Y. John Wang. Trehalose: Current use and future applications. *Journal of Pharmaceutical Sciences*, 100(6):2020–2053, 2011. ISSN 15206017. doi: 10.1002/jps.22458.

[5] Sheri L Shamblin, Lynne S Taylor, and George Zografi. Mixing behavior of colyophilized binary systems. *Journal of pharmaceutical sciences*, 87(6): 694–701, 1998.

[6] Heiko Schiffter and Geoffrey Lee. Single-droplet evaporation kinetics and particle formation in an acoustic levitator. Part 2: drying kinetics and particle formation from microdroplets of aqueous mannitol, trehalose, or catalase. *Journal of pharmaceutical sciences*, 96(9):2284–2295, 2007. ISSN 1520-6017. doi: 10.1002/jps.20858.

[7] W. W. Bromer, L. G. Sinn, A. Staub, and Otto K. Behrens. The Amino Acid Sequence of Glucagon. *Journal of the American Chemical Society*, 78 (15):3858–3860, 1956. ISSN 0002-7863. doi: 10.1021/ja01596a084.

[8] Guoqiang Jiang and Bei B. Zhang. Glucagon and regulation of glucose metabolism. *American Journal of Physiology - Endocrinology And Metabolism*, 284(4):E671–E678, 2003. ISSN 0193-1849. doi: 10.1152/ajpendo.00492.2002.

[9] Lukasz P. Kozlowski. IPC - Isoelectric Point Calculator. *Biology Direct*, 11 (1):1–16, 2016. ISSN 17456150. doi: 10.1186/s13062-016-0159-9.

[10] Cristiano Luis Pinto Oliveira, Manja Annette Behrens, Jesper Søndergaard Pedersen, Kurt Erlacher, Daniel Otzen, and Jan Skov Pedersen. A SAXS Study of Glucagon Fibrillation. *Journal of Molecular Biology*, 387(1):147–161, 2009. ISSN 00222836. doi: 10.1016/j.jmb.2009.01.020.

[11] Shirin Ghodke, Søren B Nielsen, Gunna Christiansen, Hans A Hjuler, James Flink, and Daniel Otzen. Mapping out the multistage fibrillation of glucagon. *The FEBS Journal*, 279:752–765, 2012. doi: 10.1111/j.1742-4658.2011.08465.x.

[12] Shirin D Ghodke, Anne Søndergaard, Manja A Behrens, Jan Skov Pedersen, Niels Chr Nielsen, Roland Winter, and Daniel E Otzen. *Polymorphism , Metastable Species and Interconversion : The Many States of Glucagon Fibrils.* Elsevier, 2014. ISBN 9780123944313. doi: 10.1016/B978-0-12-394431-3.00034-1.

[13] Karolina L. Zapadka, Frederik J. Becher, Shahid Uddin, Paul G. Varley, Steve Bishop, A. L. Gomes Dos Santos, and Sophie E. Jackson. A pH-Induced Switch in Human Glucagon-like Peptide-1 Aggregation Kinetics. *Journal of the American Chemical Society*, 138(50):16259–16265, 2016. ISSN 15205126. doi: 10.1021/jacs.6b05025.

[14] Marie Claire Bellissent-Funel, Ali Hassanali, Martina Havenith, Richard Henchman, Peter Pohl, Fabio Sterpone, David Van Der Spoel, Yao Xu,

and Angel E. Garcia. Water Determines the Structure and Dynamics of Proteins. *Chemical Reviews*, 116(13):7673–7697, 2016. ISSN 15206890. doi: 10.1021/acs.chemrev.5b00664.

[15] Richard Walgers, Tony C. Lee, and Arthur Cammers-Goodwin. An indirect chaotropic mechanism for the stabilization of helix conformation of peptides in aqueous trifluoroethanol and hexafluoro-2- propanol. *Journal of the American Chemical Society*, 120(20):5073–5079, 1998. ISSN 00027863. doi: 10.1021/ja973552z.

[16] Nami Hirota, Kazuko Mizuno, and Yuji Goto. Group additive contributions to the alcohol-induced α-helix formation of melittin: Implication for the mechanism of the alcohol effects on proteins. *Journal of Molecular Biology*, 275(2):365–378, 1998. ISSN 00222836. doi: 10.1006/jmbi.1997.1468.

[17] Alex Kentsis and Tobin R. Sosnick. Trifluoroethanol promotes helix formation by destabilizing backbone exposure: Desolvation rather than native hydrogen bonding defines the kinetic pathway of dimeric coiled coil folding. *Biochemistry*, 37(41):14613–14622, 1998. ISSN 00062960. doi: 10.1021/bi981641y.

[18] Stefan Grudzielanek, Ralf Jansen, and Roland Winter. Solvational tuning of the unfolding, aggregation and amyloidogenesis of insulin. *Journal of Molecular Biology*, 351(4):879–894, 2005. ISSN 00222836. doi: 10.1016/j.jmb.2005.06.046.

[19] Wojciech Dzwolak, Stefan Grudzielanek, Vytautas Smirnovas, Revanur Ravindra, Chiara Nicolini, Ralf Jansen, Anna Loksztejn, Sylwester Porowski, and Roland Winter. Ethanol-perturbed amyloidogenic self-assembly of insulin: Looking for origins of amyloid strains. *Biochemistry*, 44(25):8948–8958, 2005. ISSN 00062960. doi: 10.1021/bi050281t.

[20] Tadato Ban, Daizo Hamada, Kazuhiro Hasegawa, Hironobu Naiki, and Yuji Goto. Direct Observation of Amyloid Fibril Growth Monitored by Thioflavin T Fluorescence. *The Journal of Biological Chemistry*, 278(19), 2003. doi: 10.1074/jbc.C300049200.

[21] Jesper Søndergaard Pedersen, Dantcho Dikov, James L. Flink, Hans Aage Hjuler, Gunna Christiansen, and Daniel Erik Otzen. The changing face of glucagon fibrillation: Structural polymorphism and conformational imprinting. *Journal of Molecular Biology*, 355(3):501–523, 2006. ISSN 00222836. doi: 10.1016/j.jmb.2005.09.100.

[22] Jesper Søndergaard Pedersen, James M. Flink, Dantcho Dikov, and Daniel Erik Otzen. Sulfates dramatically stabilize a salt-dependent type of glucagon fibrils. *Biophysical Journal*, 90(11):4181–4194, 2006. ISSN 00063495. doi: 10.1529/biophysj.105.070912.

[23] W Barth. Design and layout of the cyclone separator on the basis of new investigations. *Brenn. Warme Kraft*, 8(1):9, 1956.

[24] Lingjuan Wang-Li, Calvin B. Parnell, and Bryan W. Shaw. Analysis of Cyclone Collection Efficiency. *ASAE Meeting Presentation Analysis*, 2003. doi: 10.13031/2013.15040.

[25] Héctor A. Iglesias, Jorge Chirife, and María P. Buera. Adsorption isotherm of amorphous trehalose. *Journal of the Science of Food and Agriculture*, 75 (2):183–186, 1997. ISSN 00225142. doi: 10.1002/(SICI)1097-0010(199710)75:2<183::AID-JSFA860>3.0.CO;2-T.

[26] Silvia Cardona, Carolina Schebor, María P. Buera, Marcus Karel, and Jorge Chirife. Thermal stability of invertase in reduced-moisture amorphous matrices in relation to glassy state and trehalose crystallization.

Journal of Food Science, 62(1):105–112, 1997. ISSN 00221147. doi: 10.1111/j.1365-2621.1997.tb04378.x.

[27] Andrew C Drake, Youngjoo Lee, Emma M Burgess, Jens O M Karlsson, Ali Eroglu, and Adam Z Higgins. Effect of water content on the glass transition temperature of mixtures of sugars , polymers , and penetrating cryoprotectants in physiological buffer. *PLOS ONE*, pages 1–15, 2018.

[28] Tiina Lipiäinen. *Stability and Analysis of Solid-State Forms in Pharmaceutical Powders*. Universitatis Helsinkiensis, 2018. ISBN 9789515142825.

[29] Nicole E. Hunter, Christopher S. Frampton, Duncan Q.M. Craig, and Peter S. Belton. The use of dynamic vapour sorption methods for the characterisation of water uptake in amorphous trehalose. *Carbohydrate Research*, 345(13):1938–1944, 2010. ISSN 00086215. doi: 10.1016/j.carres.2010.06.011.

[30] Nishant Kumar Jain and Ipsita Roy. Effect of trehalose on protein structure. *Protein Science*, 18(September 2008):24–36, 2009. doi: 10.1002/pro.3.

[31] Maarten A Mensink, Henderik W Frijlink, Kees Van Der Voort, and Wouter L J Hinrichs. European Journal of Pharmaceutics and Biopharmaceutics How sugars protect proteins in the solid state and during drying (review): Mechanisms of stabilization in relation to stress conditions. *European Journal of Pharmaceutics and Biopharmaceutics*, 114:288–295, 2017. ISSN 0939-6411. doi: 10.1016/j.ejpb.2017.01.024.
